# Spatially Mediated Paper Reactors for On-Site Multicoded Encryption

**DOI:** 10.1021/jacsau.4c00062

**Published:** 2024-04-22

**Authors:** Jia-Syuan Chen, Chang-Ming Wang, Po-Yu Chiang, Lee-Chiang Lo, Wei-Ssu Liao

**Affiliations:** † Department of Chemistry, National Taiwan University, Taipei 10617, Taiwan; ‡ Center for Emerging Material and Advanced Devices, National Taiwan University, Taipei 10617, Taiwan

**Keywords:** paper reactor, chemical synthesis, click reaction, encryption, on-site

## Abstract

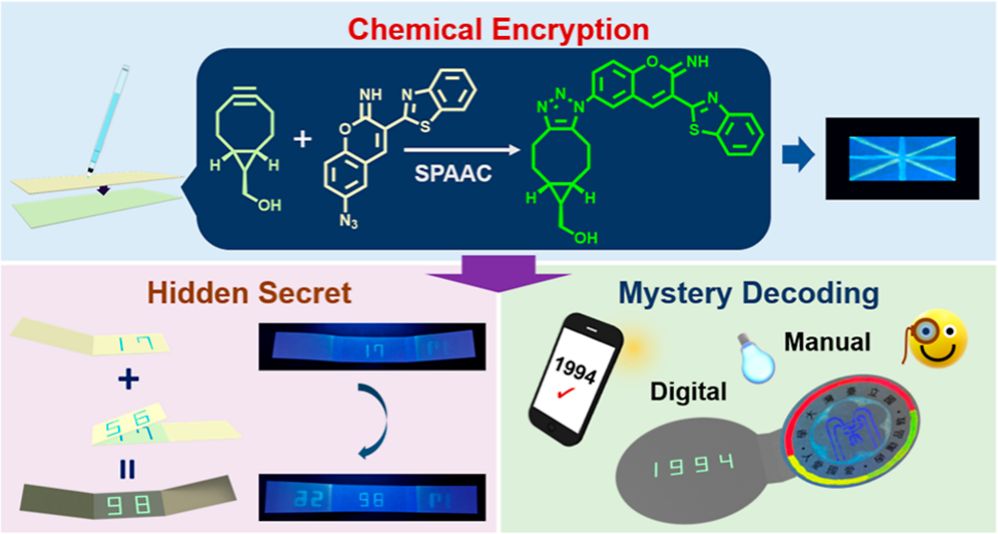

This report develops a point-of-use chemical trigger and applies it to a dual-functional chemical encryption chip that enables manual and digital identification with enhanced coding security levels suitable for on-site information verification. The concept relies on conducting continuous chemical synthesis and chromatographic separation of specified compounds on a paper device in a straightforward sketch. In addition to single-step chemical reactions, cascade syntheses and operations involving components of distinct mobilities are also demonstrated. The condensation of dione and hydrazine is first demonstrated on a linear paper reactor, where precursors can mix to react, followed by final product separation under optimized conditions. This linear paper reactor design can also support a multistep cascade Wittig reaction by controlling the relative mobility of reactants, intermediates, and final products. Furthermore, a three-dimensional paper reactor with appropriate mobile phases helps to initiate complex solvent system-driven azide–alkyne cycloaddition. By the use of a three-dimensional device design for spatially limited interdevice reactant transportation, reactants crossing designated boundaries trigger confined chemical reactions at specific positions. Accumulation of repetitive reactions leads to successful product gradient generation and mixing effects, representing a fully controllable intersubstrate chemical operation on the platform. Standing on initiating desired chemical reactions at particular interface regions, integration of appropriate selective reaction area, numerical digits overlay, color diversity, and mobile recognition realizes this dual-functional multicoding encryption process.

## Introduction

The field of chemical encryption has garnered considerable attention for its potential applications in information security and cryptography. Unlike traditional encryption methods that rely on mathematical algorithms and computational systems, chemical encryption utilizes the inherent properties of molecules to encode and decode information.^1,2^ This innovative approach combines chemical principles with information technology to provide enhanced data protection. In chemical encryption, information is encoded using specific molecules, and decryption is triggered by external stimuli such as light, temperature, magnetism, electricity, or pH changes.^3−6^ This chemically selective decryption process ensures the confidentiality and integrity of the protected message. However, there are several challenges that need to be addressed, including the synthesis of specific molecules, requirements for massive computational resources, complex processing adjustments, and limited operating conditions. To overcome these obstacles, our report aims to achieve the chemical synthesis and final product separation on a single, portable device. Success in this endeavor is expected to enable the use of paper-based devices for conducting chemical reactions, going beyond conventional analytical purposes. Additionally, we applied this approach to develop chemical encryption chips with distinctive coding capabilities, supporting both manual and digital dual-mode security confirmation. By employing different on-site chemical triggering processes on the paper substrate, we increased the complexity of the encryption and decryption steps. This advancement is particularly valuable for real-world information encryption scenarios, ensuring robust security measures.

In recent years, there has been significant interest in utilizing paper materials for the construction of portable and cost-effective devices. These materials possess numerous desirable properties, including being lightweight, inexpensive, and readily available.^7−9^ Due to the porous nature of paper, these paper-based devices also exhibit microfluidic characteristics to some extent, which brings additional benefits, such as increased throughput, reduced sample and reagent consumption, compatibility with different reaction media, and ease of operation.^10^ The combined advantages contributed to the success of the devices available today. In contrast to conventional microfluidic devices that typically use metal, glass, silicon, or polymeric materials,^11,12^ paper materials inherently possess hydrophilicity and a fibrous structure. This unique feature enables simple reagent storage and spontaneous solution transportation through capillary action, eliminating the need for external driving forces. Consequently, paper-based devices exhibit enhanced compatibility with various analytical assays and flow control techniques.^13^ Another important advantage of paper-based devices is their customizability. Hydrophobic moieties or geometries can be introduced on paper substrates by techniques such as patterning,^7,14^ printing,^15−17^ or cutting^18,19^ to create designated hydrophilic areas. This flexibility has led to the development of diverse designs for applications in analytical chemistry,^19,20^ chromatography,^21^ electrochemical detection,^22^ biomaterial and drug delivery,^23^ as well as environmental applications.^24^ Given these merits, paper-based devices are designed with simplicity of operation and rapid response in mind, making them particularly suitable for use in resource-limited settings. Overall, the combination of portable, affordable, and versatile features has garnered considerable attention and is driving the advancement of paper-based devices in various fields of practical use.

The concept of on-site generation of chemical compounds using microfluidic systems has previously been conceived, eliminating the reliance on factory or laboratory production. This approach is particularly beneficial for substances with limited shelf life or those unsuitable for transportation.^25,26^ To achieve successful on-site synthesis, it is crucial to optimize experimental parameters including controlling reactant delivery, choosing the appropriate reaction medium, and establishing an efficient product purification protocol. Microfluidic devices, due to their small-scale handling capabilities, are well-suited for meeting these demands. Furthermore, microfluidic settings can offer advantages such as increased pathway selectivity^27,28^ and reaction rates^29,30^ compared to bulk reaction environments. While microfluidic devices have demonstrated their utility in chemical synthesis, their application in chemical processing still faces challenges, particularly concerning device cost.^31^ As a result, paper-based reactors, which present advantages such as lower production costs, the ability to generate fresh chemicals on-demand, and easier manufacturing, offer a timely solution when point-of-use synthesis is required. In addition, the control of reactants can be achieved using a wide range of flow regulation and reaction environment manipulation techniques.^32,33^ In addition, when the products and reactants exhibit different mobilities on the reaction vessel, namely, chromatography paper, simultaneous separation and purification of desired products become feasible. Nevertheless, the existing paper-based devices primarily focus on sensing, analysis, and detection applications due to their convenient and direct signal reporting characteristics. Paper-based synthetic platforms are still relatively scarce in the literature.

## Results and Discussion

### Chemical Manipulations on a Paper Device

**Figure 1:**
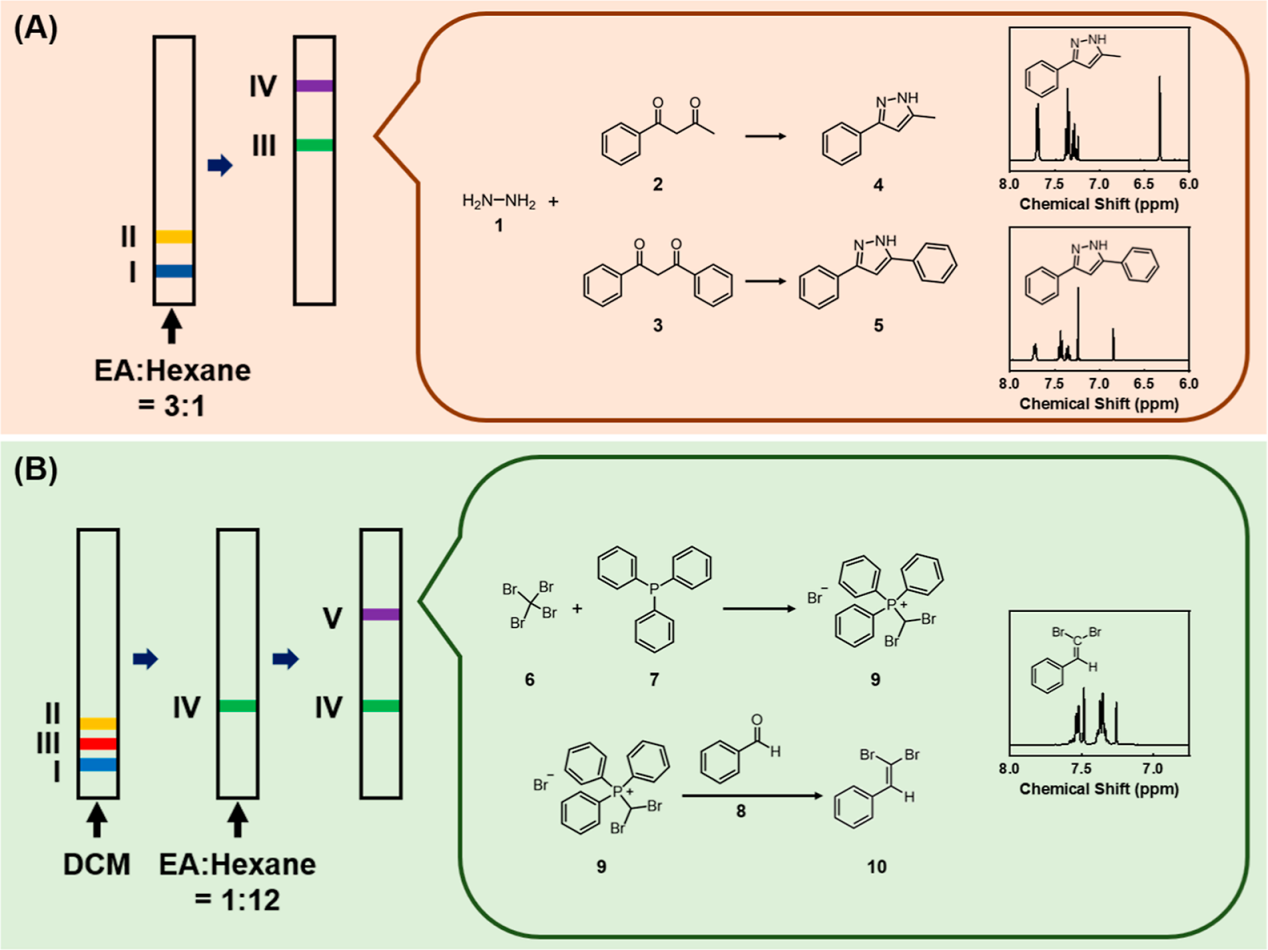
Chemical synthesis, separation, and manipulation on a paper device. (A) Paper reaction device design and the corresponding operation procedure for conducting synthesis and separation of multiple products. The reactants were initially placed at locations **I** and **II**, while desired products were generated and separated to locations **III** and **IV** after device operation. Chemical equations of the reactions performed and NMR spectra of products are also provided. Pure products without reactant residues indicate the advantageous properties of this approach. (B) Paper reaction device design and corresponding operation procedure for conducting a two-step cascade reaction and separation of products. The reactants were initially placed at locations **I**, **II**, and **III**, while the first intermediates were formed to react with the next encountering reactant at location **IV**. Further movements of carrying solvents led to the desired product separation at location **V**. Chemical equations of the reactions performed and NMR spectra of products are also provided. Pure products without reactant residues indicate the advantageous properties of this approach.

To conduct chemical reactions and achieve molecular control on a paper device, it is essential to address compatibility issues concerning reaction types, reagents, and procedures. Capillary action serves as the driving force for solution movement in the paper device, making numerous chemicals compatible with their relative mobility controlled by using solvents of appropriate polarity. Reactions that occur rapidly and yield products with direct signal reporting are particularly suitable for this setup. To explore the potential of different reactions on a paper reactor, we investigated the following three reactions: (i) condensation of dione and hydrazine, (ii) the Wittig reaction, and (iii) azide–alkyne cycloaddition. These reactions are chosen for their ability to be initiated by simple reagent mixing without the need for heating, making them ideal for proof-of-concept experiments. In addition to the chosen reactions, the proper configuration of the paper device is crucial for successful execution. The simplest form of a paper reactor is a linear design, in which reactants are spotted in sequence on the paper substrate. The reactants are carried into the mixing region and react when the solvent is introduced into the inlet. The paper substrate also functions as a chromatographic separation platform, allowing the separation of the product under the optimized conditions. To demonstrate the capability of a paper-based device as a reaction vessel for the chosen reaction type (i), we study a linear paper reactor with the initial reactant placement shown in Figure 1A. The reactions between hydrazine (**1**) and two dicarbonyl compounds, 1-benzoylacetone (**2**) and 1,3-diphenyl-1,3-propanedione (**3**), are used as examples. The reaction and separation capabilities of the platform are assessed. Prior to the reaction, a mixture of the dicarbonyl compounds **2** and **3** is spotted at location **I**, while hydrazine is spotted at location **II**. After immersing the device inlet into a solvent mixture of ethyl acetate (EtOAc)/*n*-hexane (3/1), the traveling solvent carries and mixes the spotted chemicals along the moving direction to give the products 5-methyl-3-phenyl-1*H*-pyrazole (**4**) and 3,5-diphenylpyrazole (**5**), as separated bands on the device at locations **III** and **IV**.

Building on the successful synthesis and purification of chemicals on a paper-based device, we investigated a more complex cascade chemical operation (Figure 1B). The paper reactor is tailored to perform multistep cascade reactions through careful placement of reagents. Understanding the relative mobility of reactants, intermediates, and final products becomes crucial in planning the initial reactant positions and corresponding eluents. In this demonstration, a linear paper-based reactor is employed to showcase the potential for cascade reactions in the context of a Wittig reaction [reaction type (ii)]. The Wittig reaction involves three reactants: CBr_4_ (**6**), triphenylphosphine (**7**), and benzaldehyde (**8**). These reactants are placed in sequence on the linear reactor at locations **I**, **II**, and **III**, respectively. CH_2_Cl_2_ is used as the carrying solvent. Due to differences in mobility, CBr_4_ moves faster than benzaldehyde when eluted with CH_2_Cl_2_, leading it to react with PPh_3_ first. This results in the formation of the intermediate (dibromomethyl)(triphenyl)phosphonium bromide (**9**), which remains immobile under elution with CH_2_Cl_2_ and is found at the location **IV**. When benzaldehyde arrives at the location **IV**, it reacts with intermediate **9** to produce the final product, 2,2-dibromostyrene (**10**). To separate 2,2-dibromostyrene from side reaction products and leftover reactants, the mobile phase is changed to a solvent mixture of EtOAc/*n*-hexane (1/12). Upon replacement of the solvent, 2,2-dibromostyrene is carried further down the device to location **V**. This demonstration illustrates that with thoughtful planning based on the physical and chemical properties of the reactants, it is possible to conduct chemical reactions involving multiple steps.

It is also interesting to note that the dimensions of a linear paper reactor are crucial in controlling the chemical reactions. To illustrate this, paper reactors with identical lengths but different widths are used to conduct the condensation reaction of 1-benzoylacetone and hydrazine (Figure S1). An equal amount of the two reactants are spotted at positions **I** and **II**, respectively, while the mixture of EtOAc and *n*-hexane (3/1) is used as the mobile phase. Under the same reaction period, product quantities generated from these paper reactors are found to be 0.14 mg (2 cm-wide strip), 0.25 mg (1 cm-wide strip), and 0.42 mg (0.5 cm-wide strip). This indicates a faster reaction rate when a smaller reactor channel dimension is used to operate chemical reactions in this system.

### Building Three-Dimensional Paper Reactors

**Figure 2:**
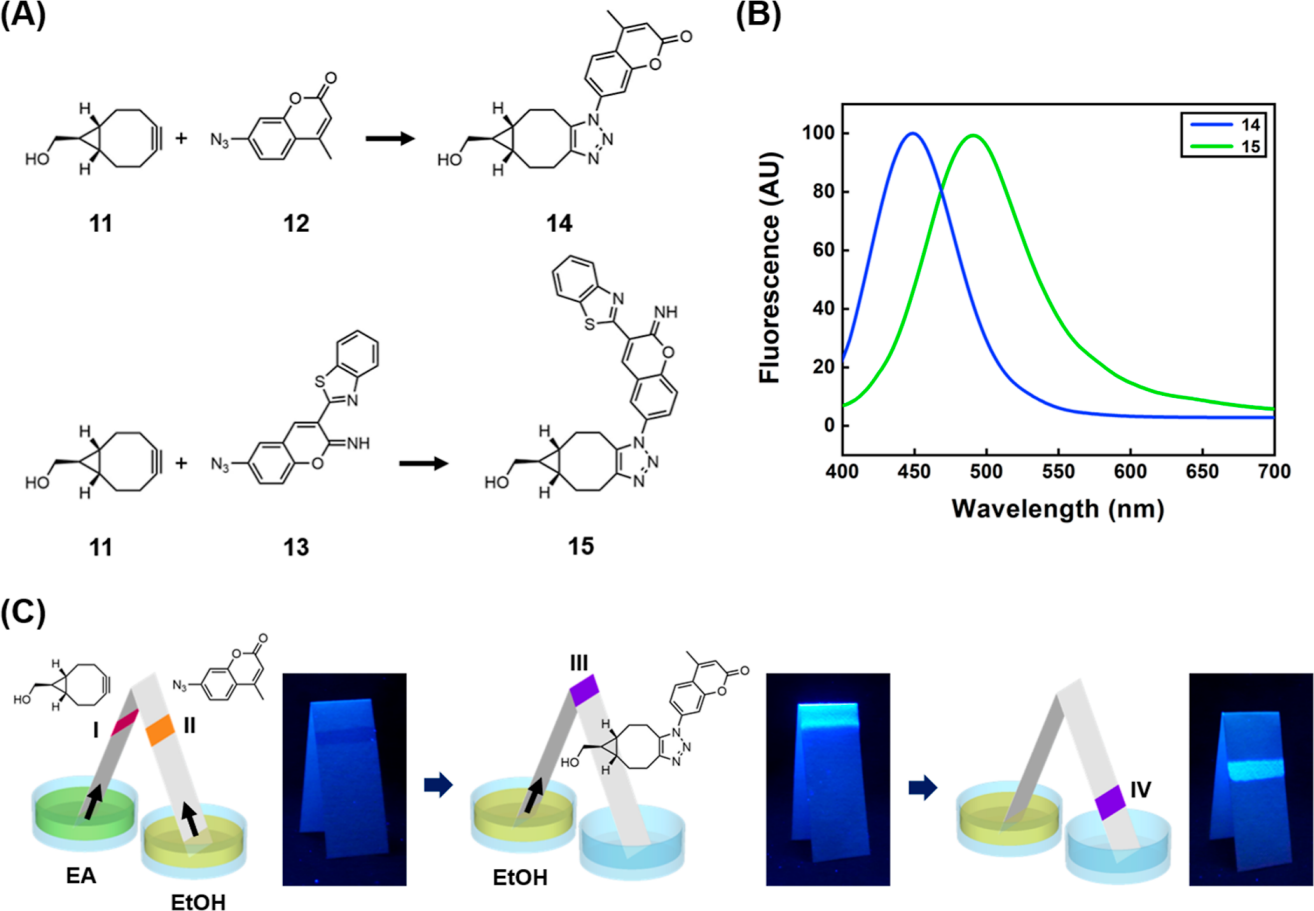
(A) Strategy of SPAAC by using BCN (**11**), AzMC (**12**), and AzBTCI (**13**) as the click reaction partners. (B) Fluorescence spectra of two different azido-containing fluorogenic compounds (**14**) and (**15**) produced from processes in (A). (C) Paper reaction device design and the corresponding operation procedure for conducting reaction between species in distinct solvents and the migration of products. The reactants were initially placed at locations **I** and **II**, while desired products were generated at location **III**. The click reaction resulting fluorescent traces could be observed before and after movement (**IV**) under 365 nm of UV illumination.

The choice of solvent often plays a crucial role in the reaction conversion and yield of chemical synthesis. Chromatographic paper, with its porous structure, enables the transportation of various solvents, making it ideal for manipulating reactants in solvents with different polarities. As a demonstration of reaction type (iii), a click reaction utilizing the strain-promoted azide alkyne cycloaddition (SPAAC) strategy is conducted, in which ((1*R*,8*S*,9r)-bicyclo[6.1.0]non-4-yn-9-yl)methanol (**11**, BCN)^34^ acts as the strained cycloalkyne component (Figure 2A). Meanwhile, two different azido-containing fluorogenic compounds, 7-azido-4-methylcoumarin (**12**, AzMC)^35^ and 6-azido-3-(benzo[*d*]thiazol-2-yl)-2*H*-chromen-2-imine (**13**, AzBTCI), are used as the click reaction partners. Both **12** and **13** show minimal fluorescence due to the quenching effect of the azido group.^36^ However, when reacted with **11**, they form triazole derivatives **14** and **15**, which are highly fluorescent with emissions at 449 and 491 nm, respectively (Figure 2B). This fluorescence turn-on property thus becomes a useful reporter for monitoring the reaction progress on a paper device. The experimental setup employs a bridge-shaped paper reactor, supporting chemical reactions with reactants dissolved in solvents of different polarities (Figure 2C). BCN **11** is carried by EtOAc from the left arm’s location **I**, while AzMC **12** is carried by ethanol from the right arm’s location **II**. The reaction between these two reactants is initiated when the solvent front meets at the middle of the paper device (location **III**). The confined space in paper fibers and continuous capillary pressure from both ends enable the reaction to proceed without additional mixing steps. To explore the regulation of product movement in paper-based devices, this paper reactor is temporarily removed from the solvent reservoir and the solvents are allowed to evaporate. Ethanol is then used to elute from the BCN side (left arm). The resulting fluorescent product is mobile in ethanol and moves toward the location **IV**. Hence, chemical synthesis under complex solvent systems can be efficiently conducted using this simple paper-based reactor. Moreover, careful selection of mobile phases not only aids in completing chemical reactions involving different solvents but also allows for the manipulation of chemical movements on a three-dimensional paper reactor.

### Controlled Chemical Reactions by Interdevice Boundary Crossing

**Figure 3:**
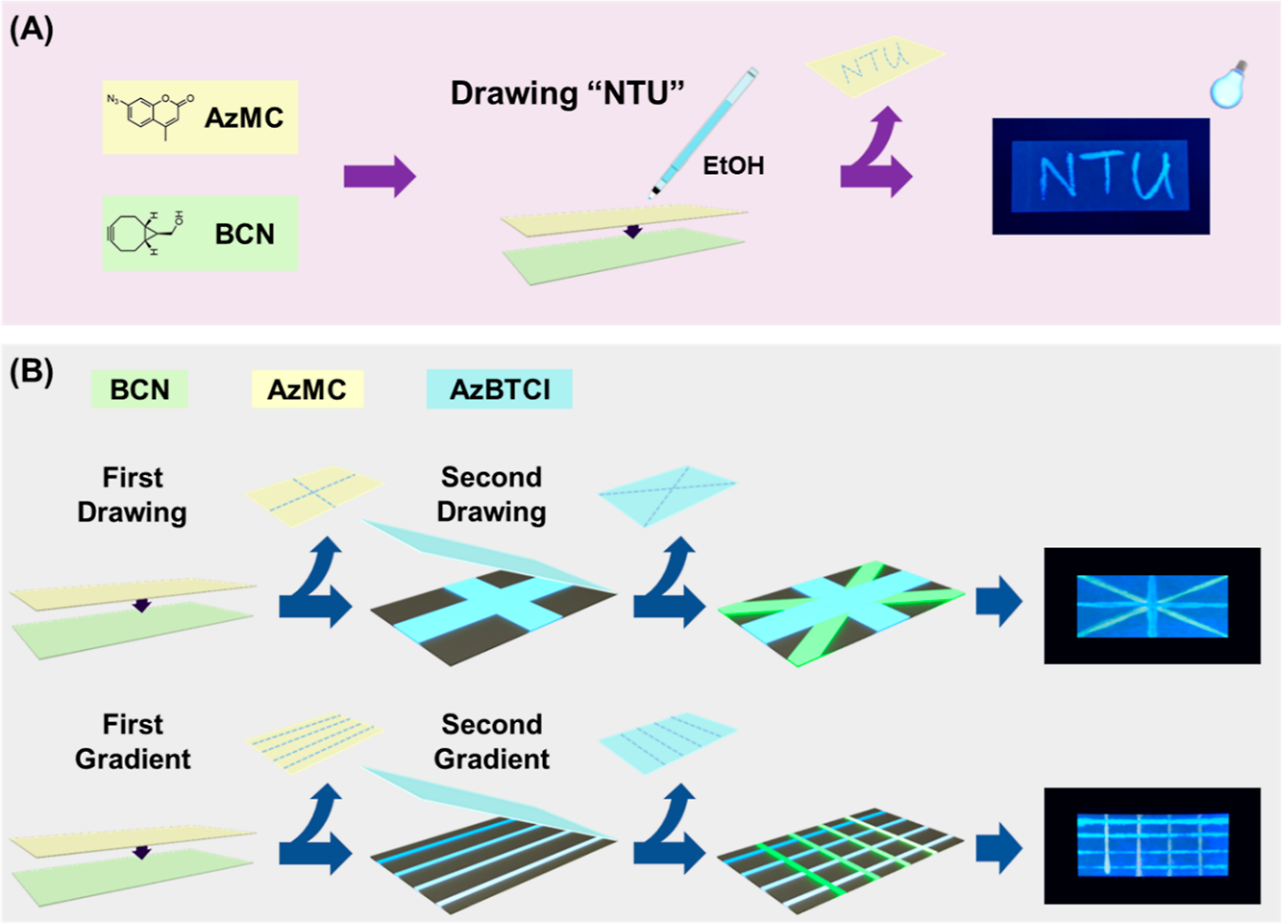
Conducting three-dimensionally controlled chemical reactions by interdevice boundary crossing. (A) Two filter paper pieces were soaked with BCN **11** and AzMC **12** independently, dried, and overlaid together. An ethanol-delivering pen was then used to directly write arbitrary features on top of the assembled device. After writing, the two paper pieces were immediately separated, and the SPAAC click reaction-resulting fluorescent pattern could be observed at the bottom piece under 365 nm of UV illumination. (B) Multilayered click reactions on a paper device through consecutive writings with AzMC/BCN and AzBTCI/BCN pairs. Top raw: integrated cross drawings produced a fluorescent Union Flag shape. Bottom row: repetitive drawings on the device led to a color gradient, and the orthogonal operation resulted in the final fluorescent gradient matrix.

Expanding the concept of stereoscopic operations on a paper device, the paper reactor can be further developed to accommodate intricate configurations, allowing for the creation of practical devices with diverse functions without the need for complex processing. This idea relies on transporting reactants across device boundaries to initiate chemical reactions that are spatially confined to specific regions of encounter. By overlapping paper substrates impregnated with individual reactants and introducing transport solvents, the movement of chemicals can be controlled and the corresponding reaction process is confined within the region where the reactants meet. In a practical test (Figure 3), we prepare two pieces of filter paper, each dipped into separate solutions of BCN **11** and AzMC **12**. After drying at room temperature, we stacked the two filter papers together, with the AzMC paper on top and the BCN paper on the bottom. Using a solvent (ethanol) delivery pen, we directly write characters on the AzMC paper and immediately separate the two sheets. The fluorescent turn-on property of the AzMC molecule after the writing initiates a cycloaddition reaction, leading to fluorescent writing marks “NTU” appearing on the bottom sheet under 365 nm of UV illumination (Figure 3A). This successful boundary crossing transportation of reactants between the two layers of paper demonstrates the feasibility of this approach. The writing-triggered chemical reaction retains its feature integrity due to the limited quantity of reactants involved. Importantly, no additional prepatterning or fluidic channel setup is required in this operation. The delivery of reactants and the initiation of reactions can be arbitrarily programmed according to user needs. Various types of paper materials are tested, all of which can produce comparable results with slight outcome differences (Figure S11A–F). Thinner filter paper facilitates easier solvent penetration to achieve equivalent results using a less amount of reaction carrying solvent. Besides, smaller paper pore size and higher surface hydrophobicity induce obvious solvent lateral diffusion and diminish pattern resolution. These results indicate that the paper material permeability affects the reagent transportation behavior when crossing an interface, thereby controlling the reaction levels within confined spaces. Insufficient reactant delivery quantities or incomplete reaction progress may lead to a faint visualization color and inaccurate result interpretation.

The advantage of this design becomes even more apparent when creating multilayered chemical reactions on a single paper piece (Figure 3B). Utilizing a similar alkyne azide cycloaddition, three paper substrates soaked with BCN **11**, AzMC **12**, and AzBTCI **13** are prepared. The BCN piece serves as the bottom support, followed by the assembly of the AzMC sheet on top of it. Using the ethanol-carrying writing pen, we first drew a cross on the two-layer device. The AzMC sheet is then replaced with the AzBTCI sheet, and a second pen stroke of a diagonal cross is performed. The bottom support paper is then observed under 365 nm UV illumination, and both traces are clearly visible, forming a fluorescent Union Flag shape. Interestingly, the crossover region exhibits a robin’s egg blue color, indicating the additive property of chemical reactions on this platform. Meanwhile, the ability to achieve multistaged chemical operations on a single support is showcased. Each drawing constrains the delivery of a specific quantity of reactants, enabling control over the extent of chemical reactions that are transferred between the substrates. By repeating drawings at designated positions on the paper device, we can control and adjust the extent to which chemical reactions can be controlled and adjusted. As displayed in Figure 3B, two perpendicular sets of parallel lines with different numbers of repetitive drawings create a color gradient based on the accumulation of reaction products. This leads to a final gradient color mixing matrix, suggesting a fully controllable intersubstrate chemical operation on the platform.

### On-Site Paper Encryption Chip Designs

**Figure 4:**
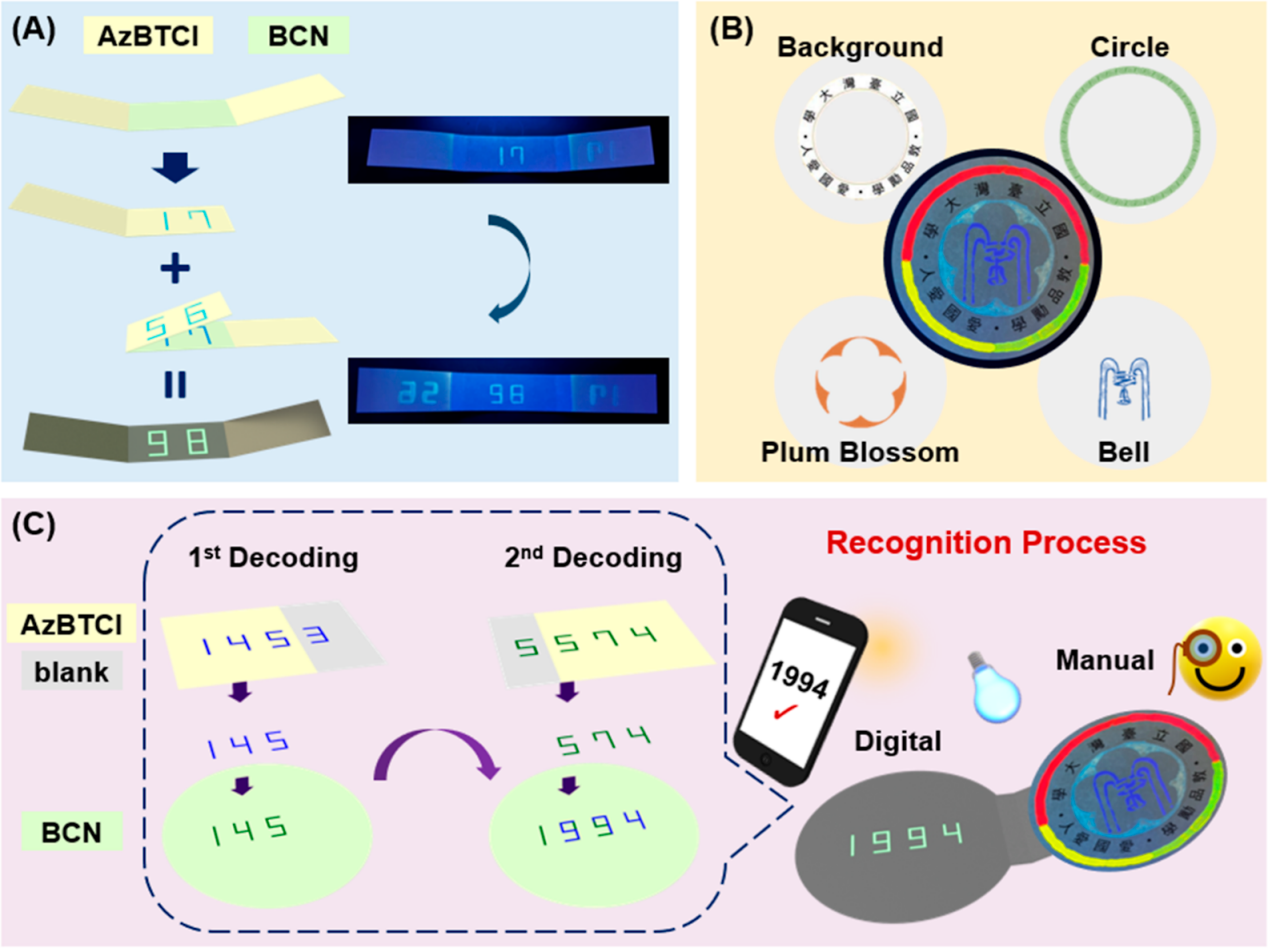
Design principles of an on-site paper encryption chip. (A) Intersubstrate coding integration strategy. A paper substrate was divided into three foldable regions soaked with AzBTCI **12** (left and right) and BCN **11** (center), independently. The sequential fold-and-draw procedures generated distinct reaction-dependent code numbers in the central region, and a final integrated code was observed under 365 nm UV illumination. (B) Multifluorescence imaging alignment approach. A paper substrate preprinted with black features was first soaked with BCN **11** and acted as the following reaction platform. The red/green/yellow tricolored circle was created by capillary tube direct dye writing on this paper piece. Drawing-induced click chemistry produced a fluorescent bell shape and plum blossom pattern on the platform when a AzBTCI and a AzMC soaked layer were consecutively overlaid for reaction. (C) Dual-functional encryption device containing both manual (right) and digital check (left) parts. The design principle of penta-fluorescent color painting/black feature background on the right part is the same as that illustrated in (B) for a manual checking purpose. The digital check part is composed of two serial decoding processes using featureless AzBTCI-coated sheets. These two decoding sheets entail specific regional AzBTCI coating and can therefore initiate reactions only at particular locations of the bottom BCN layer. In the first decoding process, only code “145” was transferred onto the underneath layer when code “1453” was written. Similarly, writing code “5574” only produced code “574” at the bottom layer in the second decoding step. Therefore, the correct final code “1994” was produced, and the fluorescent pattern could be digitally confirmed by a smartphone (Video S1).

We envision that a strategy exploiting multiple chemical reactions with additive properties on a paper piece will facilitate the development of more complex devices for practical applications, such as multicoded encryption chips. To achieve this intent, we incorporate two strategic modes on a paper piece to boost its encoding capabilities: intersubstrate coding integration and multifluorescence imaging alignment. In the first encryption mode, a sequential fold-and-draw procedure is employed to produce the integrated code through two intersubstrate chemical reactions (Figure 4A). A fibrous paper is divided into three sections with AzBTCI **13** loaded at both ends and BCN **11** loaded in the middle region. The AzBTCI region on one side is folded inward to cover the middle BCN position, and the number “17” is written using an ethanol-carrying pen. After unfolding, the other AzBTCI region is folded inward to cover the middle BCN position, and the number “56” is written. Unfolding the overlaid section reveals a fluorescent “98” character at the middle section. This fold-and-draw approach demonstrates consecutive stacking of fluorescent chemical reaction products and validates the feasibility of intersubstrate transportation for integrated coding. In the second encryption mode, multilayer chemical reaction alignments are performed on an assembled paper device for multicolor fluorescence imaging. As illustrated in Figure 4B, a fluorescent multicolored NTU emblem pattern is created from four consecutive encoding processes. A BCN paper with preprinted black features and a red/green/yellow tricolored circle serves as the reaction platform. Using an ethanol-carrying pen, the drawing of a bell object is applied on an overlaid AzMC paper sheet. Following the same process, the plum blossom pattern is drawn using an AzBTCI paper as the overlaid sheet. This results in a penta-fluorescent color painting on a black feature background on the reaction platform. The successful combination of multicolor reaction products on the same paper substrate confirms the delicate chemical controls of three-dimensional boundary crossing transportation in this approach. Utilizing these two encryption designs, a multicoded chip is demonstrated in Figure 4C, with manual checks on the right and digital recognition on the left part. In a real operation (Video S1), manually inspecting the fluorescent patterns on the right of six similar-looking chips screens out two candidate chips. The comparison between the standard sample and the tested chip allowed staff to manually verify the authenticity of a piece. Afterward, the left-hand side of the two selected chips is sequentially overlaid with two decoding sheets, and an ethanol delivery pen is used for the consecutive code number drawing. Interestingly, the identical dual-code drawing produces different integrated numbers on each chip under UV light illumination (Video S1). This is due to a pre-encryption step on the overlaid featureless decoding sheets (see figure caption for design details) through selective regional AzBTCI coating. This approach enhances the security levels of a decoding sheet, and fluorescent number pattern recognition is accomplished and digitally verified by a smartphone. It should also be noted that the on-site fluorescence turn-on property of selected click reaction reactants serves as a valuable indicator for monitoring the decoding progress on a paper device. This feature mitigates the influence of side reactions or interference species, thereby ensuring robustness and reliability of the decoding process identification. Therefore, special attention should be given to the design of fluorescent molecules, the reactivity of reactions, the generation of byproducts, and color overlay blending with other factors, when a similar decryption concept is adopted.

## Conclusions

In conclusion, we have successfully demonstrated the straightforward chemical synthesis, purification, and manipulation of organic compounds on a paper-based platform. The capillary force allows various solvents to move spontaneously and carry reactants along the device, initiating designated reactions at specific locations. The chromatographic nature of the paper device also allows us to separate products from excess reactants based on their mobility differences. The versatility of our approach enables the achievement of multiplexed single-step reactions, two-step cascade reactions, and reactions occurring between distinct polarity environments. By carefully controlling the movement of reactants, intermediates, and products, we can trigger the desired chemical processes at specific locations, facilitating subsequent operations and fine chemical collection. This achievement extends to delicate chemical operations on three-dimensional paper reactors, where interdevice boundary transport of reactants in multilayered designs allows for precise spatial controls of reactions at specified interfaces. Taking advantage of this approach, we can conduct on-site chemical reactions tailored to specific needs and apply them to dual-functional chemical encryption chips. These chips integrate both manual and digital recognition processes on the same device, providing a practical design for authentic chip verification and remote recognition capabilities. The chemical encryption chips significantly enhance the security levels of the decoding process, offering a new perspective on designing information guardians for on-site verification purposes. Based on these achievements, the presented interface reaction controlling strategy holds great potential not only in lab-on-chip device applications but also in advancing current chemical operation capability. For example, unique platforms supporting an interface-mediated crystal growing system, signal-enhanced spectroscopic technique, promoted microbial detection/inhibition, and delicate nanomaterial synthesis are expected. These directions should differentiate from conventional areas and give opportunities warranted by the special interface-limited chemical environment.

## Experimental Section

### Materials and Chemicals

Advantec chromatography filter paper was obtained from Advantec (Tokyo, Japan). Kraft paper was obtained from IKEA (Leiden, The Netherlands). Kimwipes was obtained from Kimberly-Clark Corporation (Irving, TX, USA). Natural hemp paper was obtained from Mentholatum (Orchard park, NY, USA). Washi paper was obtained from Sanrio (Tokyo, Japan). 1-Benzoylacetone, hydrazine monohydrate, tetrabromomethane, benzaldehyde, and triphenylphosphine were purchased from Acros Organics (Pittsburgh, PA, USA). 1,3-Diphenyl-1,3-propanedione was purchased from Alfa Aesar (Ward Hill, MA, USA). EtOAc, ethanol, *n*-hexane, dichloromethane, and deuterated chloroform were purchased from Echo Chemical (Miaoli, Taiwan).

### Simultaneous Reaction and Separation of Multiple Products

1-Benzoylacetone (66 mM in ethanol, 20 μL) and 1,3-diphenyl-1,3-propanedione (66 mM in ethanol, 20 μL) were spotted on a strip of chromatography paper (10 cm long and 2 cm wide) 1.5 cm from the inlet end, while hydrazine (66 mM in ethanol, 40 μL) was spotted 2 cm from the inlet end. (The spotting locations of reagents were pretested through thin layer chromatography.) The amount of hydrazine solution spotted was equal to that of 1-benzoylacetone and 1,3-diphenyl-1,3-propanedione combined, resulting in equal equivalents. The inlet end of the chromatography paper was immersed in a mixture of EtOAc and *n*-hexane (3/1) for 7 min until the solvent front reached 6.5 cm from the inlet end to allow the solvent to mix reactants located at different positions. After the solvent evaporated and solid precipitation occurred, appropriate sections of the paper device were cut out and placed in CDCl_3_ to extract the reaction products. These product-containing solutions were then analyzed with ^1^H NMR spectroscopy (Varian 400 MHz-NMR, Palo Alto, CA, USA), demonstrating the successful synthesis and efficient product separation of reactions on the paper device.^37,38^ The quantities of 5-methyl-3-phenyl-1*H*-pyrazole (**4**) and 3,5-diphenylpyrazole (**5**) products were determined to be 0.13 and 0.15 mg, respectively.

### Two-Step Cascade Reactions

On a strip of chromatography paper (10 cm long and 2 cm wide), tetrabromomethane (0.25 M in dichloromethane, 20 μL), benzaldehyde (0.17 M in dichloromethane, 20 μL), and triphenylphosphine (0.5 M in dichloromethane, 20 μL) were spotted at 1, 1.5, and 2 cm from the inlet end, respectively. (The spotting locations of reagents were pretested through thin layer chromatography.) The inlet end was immersed in dichloromethane for 2 min until the solvent front reached 4 cm from the inlet end, after which the device was removed from the dichloromethane reservoir and left to dry. Thereafter, the device inlet was immersed in *n*-hexane for 7 min until the solvent front reached 6.5 cm. After solvent evaporates and solid precipitation forms, the reaction product band on the device is cut out and then extracted with CDCl_3_, followed by analysis with ^1^H NMR spectroscopy.^39^ The quantity of the products is determined to be 0.31 mg.

### Paper Reactor Dimension Effect

Three chromatography paper strips with the same length (10 cm) but different widths (2, 1, and 0.5 cm) were used in this test. 1-Benzoylacetone (66 mM in ethanol, 20 μL) and hydrazine (66 mM in ethanol, 20 μL) were spotted at 1 and 2 cm from the inlet end, respectively. (The spotting locations of the reagents were determined through pretesting via thin layer chromatography). The inlet end of the chromatography paper was immersed in a mixture of EtOAc and *n*-hexane (3/1) for 7 min until the solvent front reached 7 cm from the inlet end, allowing for the mixing of reactants located at different positions. Following solvent evaporation and solid precipitation, sections of the paper device containing reaction products were cut out and placed in CDCl_3_ for extraction. These extracted solutions were then analyzed by using ^1^H NMR spectroscopy (Varian 400 MHz-NMR, Palo Alto, CA, USA).

### Reaction and Movement of Chemicals from a Multisolvent System

BCN (**11**, 11.7 mM in EtOAc, 20 μL) was spotted on a strip of chromatography paper (10 cm long and 2 cm wide) 4 cm from one end, while AzMC (**12**, 11.7 mM in ethanol, 20 μL) was spotted 4 cm from the other end. The BCN end and the AzMC end of this chromatography paper were simultaneously placed in bulk EtOAc and bulk ethanol solvents, respectively. After fluidic fronts meet at the device center and allowed 5 min of incubation, the device was removed from the solvent reservoirs and left to dry. Thereafter, the BCN end of the chromatography paper was placed in ethanol for 10 min, which moves the fluorescent products toward the other end, and its trace can be monitored under 365 nm of UV illumination.

### Three Dimensionally Controlled Interdevice Chemical Reaction

In a two-layer device design, two chromatography papers (5 cm long and 2 cm wide) were independently soaked with BCN (**11**, 11.7 mM in EtOAc) and AzMC (**12**, 11.7 mM in ethanol). After drying and overlaying the two pieces together with AzMC paper on the top and BCN paper at the bottom, an ethanol-delivering pen was used to directly write “NTU” character on top of the assembled device. After the two paper pieces were separated, a fluorescent character could be observed at the bottom piece under 365 nm of UV illumination. When different paper types were tested, identical operation was applied. In a multilayer device design, three chromatography papers (5 cm long and 2 cm wide) were independently soaked with BCN (**11**, 11.7 mM in EtOAc), AzMC (**12**, 5.5 mM in ethanol), and AzBTCI (**13**, 5.5 mM in DCM). After drying these paper pieces, two of these pieces were selectively assembled together based on requirements.

The fluorescent Union Flag shape was created by drawing a cross on the two-layer device (with AzMC paper on the top and BCN paper at the bottom) with the ethanol-carrying pen, while the AzMC sheet was thereafter replaced with the AzBTCI sheet, and a secondary diagonal cross drawing was applied. After the AzBTCI sheet was removed, the bottom support paper was monitored under 365 nm of UV illumination, revealing a Union Flag shape. To create the color gradients, a set of parallel lines with two, four, six, and eight repetitions was first generated on the two-layer device (with AzMC paper on the top and BCN paper at the bottom). By rotating the device at 90°, the AzMC sheet was replaced with an AzBTCI sheet, and a set of parallel lines with two, four, six, and eight times of repetitive drawings were again generated. This produced color gradients perpendicular to each other when the bottom support paper was monitored under 365 nm UV illumination.

### Fabrication of the On-Site Paper Encryption Chip

The intersubstrate coding integration strategy was realized by dividing a chromatography paper (15 cm long and 2 cm wide) into three foldable regions (5 cm each). The left and right regions were independently soaked with AzBTCI (**13**, 5.5 mM in DCM), while the central region was soaked with BCN (**11**, 11.7 mM in EtOAc). When the right side of the paper was folded, with the AzBTCI-soaked part on top of the BCN-soaked section, using an ethanol-carrying pen to write the character “17” generated “17” in the central region. Afterward, a similar operation was applied when the right side was unfolded and the left side was folded instead, and the character “56” was created at the same region. This integral writing led to the appearance of a character “98” at the bottom support paper at the end under 365 nm of UV illumination.

To create the multifluorescence imaging alignment, circular filter papers (9 cm in diameter) preprinted with black features were soaked with BCN (**11**, 11.7 mM in EtOAc). A capillary tube was then used to directly transfer BODIPY (1 M in methanol), NBD (1 M in methanol), and sulforhodamine B (1 M in methanol) dyes to the BCN-soaked paper for generating the tricolored circle. Thereafter, the bell object and plum blossom pattern were created by sequential overlaying and drawing with AzMC-soaked (5.5 mM in ethanol) and AzBTCI-soaked (5.5 mM in CH_2_Cl_2_) paper sheets. The final bottom support paper was monitored under 365 nm of UV illumination. [Note: this multifluorescent pattern remains after one year of storage in ambient, representing the long shelf life of the reaction products generated on this platform (Figure S12).]

In the dual-functional encryption device design, two encryption sections were created on a single device, including manual check (Video S1, 0:00–1:15) and digital-recognition (Video S1, 1:15–3:00) parts. The design principle of the manual check part was identical to the aforementioned multifluorescence imaging alignment, where multiple optical patterns are created for the authentic chip manual check purpose. On the other hand, the design of the digital-recognition part was similar to the intersubstrate coding integration strategy describe above. In this operation, the circular paper part (9 cm in diameter) was presoaked with BCN (11.7 mM in EtOAc) acting as the bottom layer and two rectangular paper sheets (5 cm long and 2 cm wide) presoaked with AzBTCI (5.5 mM in DCM) could thereafter be overlaid as decoding sheets. It should be noted that only certain portions of the rectangular sheets were precoated with AzBTCI (Figure 4C) and were the only places that could initiate the click reaction. This allowed the selective decoding process when the first code “1453” and the second code “5574” were integrated into the correct code “1994” on the bottom layer (Video S1, 1:15–2:00). Notably, an incorrect code “9999” would be generated when the whole rectangular decoding paper sheets were presoaked with AzBTCI (Video S1, 2:00–3:00). This digital-recognition process could be accomplished by a smartphone when the device was operated under 365 nm UV illumination.

## Supporting Information Available

Paper reactor dimension effect, synthesis of AzBTCI (**13**), ^1^H NMR spectrum of compound **S2** (400 MHz, CDCl_3_), ^1^H NMR spectrum of compound **S4** (400 MHz, CDCl_3_), ^1^H NMR spectrum of compound **S5** (400 MHz, CDCl_3_), ^1^H NMR spectrum of compound **13** (400 MHz, DMSO-*d*_6_), ^13^C NMR spectrum of compound **13** (100 MHz, DMSO-*d*_6_), ^1^H NMR spectrum of compound **14** (400 MHz, CDCl_3_), ^13^C NMR spectrum of compound **14** (400 MHz, CDCl_3_), ^1^H NMR spectrum of compound **15** (400 MHz, CDCl_3_), ^13^C NMR spectrum of compound **15** (100 MHz, DMSO-*d*_6_), effects of different paper types, and shelf life test of the reaction products (PDF)
The decoding process of a dual-functional encryption chip (AVI)

## Supplementary Material

au4c00062_si_001.pdf

au4c00062_si_002.avi
