# Supplementary material for: Spatially Mediated Paper Reactors for On-Site Multicoded Encryption
Source: JACS Au. 2024 Apr 22;4(6):2151–9. doi: 10.1021/jacsau.4c00062 (PMC11200220; doi:10.1021/jacsau.4c00062)
Supplement: Supplementary file 1 — au4c00062_si_001.pdf [file au4c00062_si_001.pdf]

Supporting Information

Spatially Mediated Paper Reactors for On-Site Multicoded Encryption

Jia-Syuan Chen,[1] Chang-Ming Wang,[1] Po-Yu Chiang,[1] Lee-Chiang Lo,[1] and Wei-Ssu Liao[1,2]**

[1] Department of Chemistry, National Taiwan University, Taipei 10617, Taiwan

[2] Center for Emerging Material and Advanced Devices, National Taiwan University, Taipei 10617, Taiwan

*To whom correspondence should be addressed: wsliaochem@ntu.edu.tw (W.S.L.); lclo@ntu.edu.tw

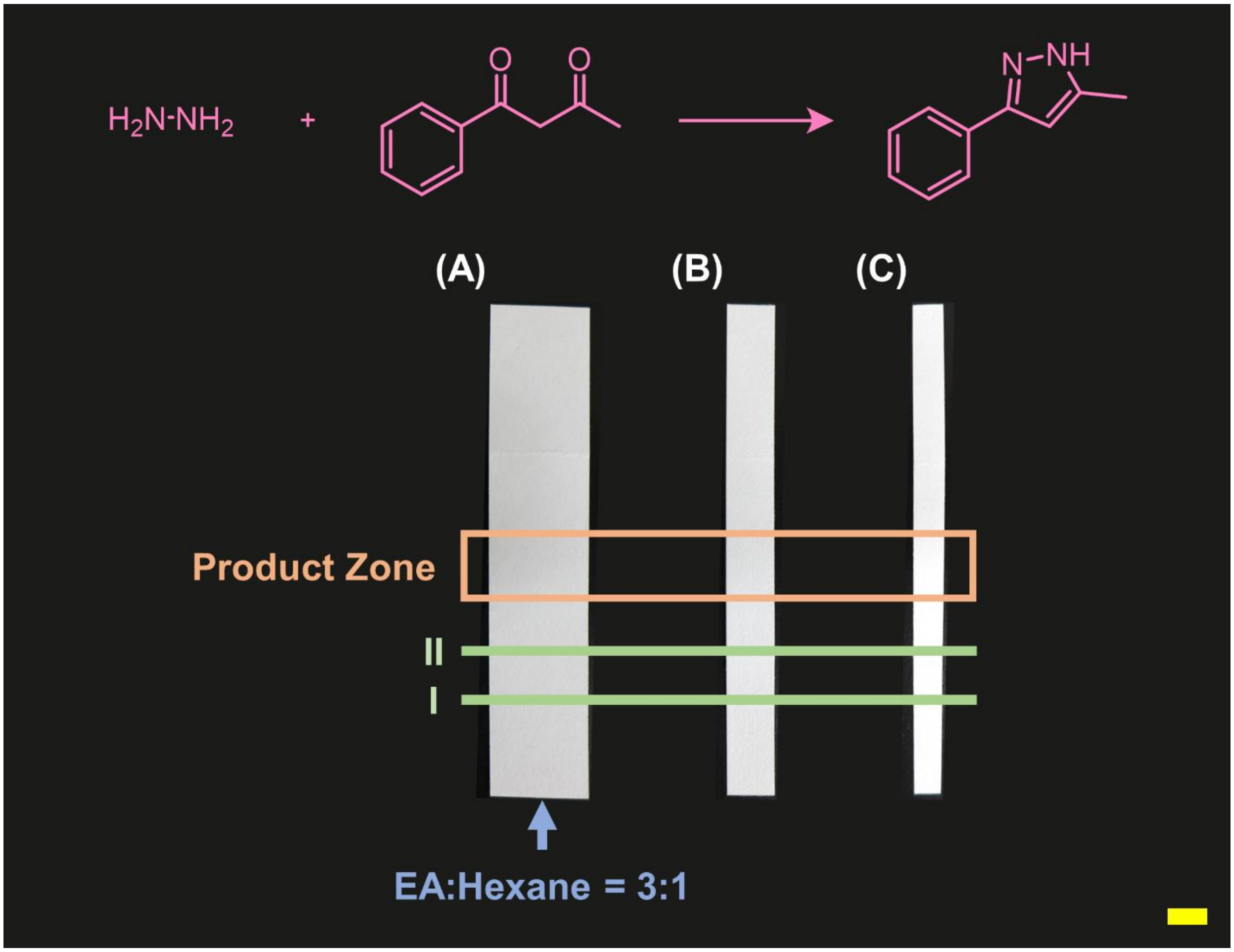

Figure S1. Paper reaction device design and corresponding operation procedure for paper reactor dimension effect tests. (A) 2 cm-wide strip, (B) 1 cm-wide strip, and (C) 3 cm-wide strip. The scale bar is 1 cm.

Reagents and Instruments

All reagents and starting materials were obtained from commercial suppliers (Acros, Aldrich and Merck) and were used without further purification. Pyridine, dichloromethane, and acetonitrile were distilled from calcium hydride.

Analytical TLC (silica gel, $60F_{254}$, Merck) were visualized under UV light or stained with phosphomolybdic acid-ethanol (PMA), potassium permanganate (5% $KMnO_4$ in 1M NaOH with 5% potassium carbonate) and ninhydrin (1.5% in 3% acetate acid with *n*-butenol solution) or iodine. Column chromatography was prefomed with Kiesegel 60 (230-400 mesh) silica gel (Merck). Mobile phases are reported in ratio of solvents for binary systems (*e.g*. EtOAc/hexane = 1/4).

Nuclear magnetic resonance (NMR) spectra were recorded on either Bruker AVIII or Bruker DPX (^{1}H: 400 MHz; ^{13}C: 100 MHz) spectrometer. All chemical shifts are quoted on the δ scale in ppm using residual solvent as the internal standard (^{1}H NMR: $CDCl_3$ = 7.26, MeOD-d_4 = 3.31, DMSO-d_6 = 2.50, D_2O = 4.79 and ^{13}C NMR: $CDCl_3$ = 77.2, MeOD-d_4 = 49.0, DMSO-d_6 = 39.5). Coupling constant (J) are reported in hertz (Hz) with the following abbreviations: s = singlet, d = doublet, t = triplet, q = quartet, m = multiplet, b = broad. Raw FID files were processed by Bruker TopSpin 4.0 or Bruker TopSpin 3.6 software.

Infrared spectrospcopy (IR) spectra were recorded on a Varian 640 FT-IR instrument by applying the sample onto NaCl plate (neat) or prepared it into KBr plate (KBr), and the absorption data are reported in cm^{-1}. Melting points were recorded using Fischer Scientific melting point apparatus and all samples are recorded without correction. Electrospray ionization high resolution mass spectrometry (ESI-HRMS) data were obtained using Waters LCT Premier XE instrument.

Experiment Sections

Synthesis of AzBTCI (13)

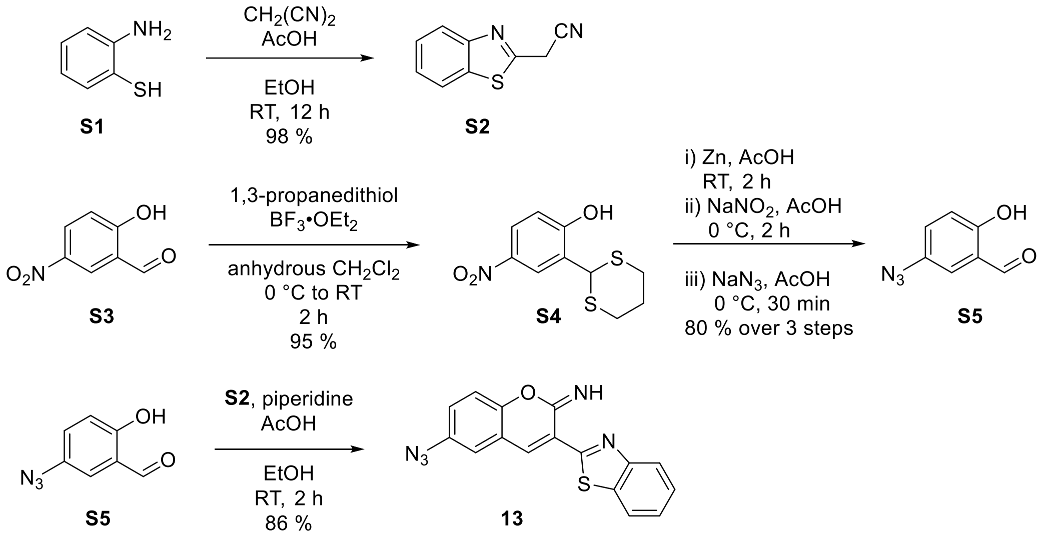

Scheme S1. Synthesis of AzBTCI (**13**).

2-(Benzo[d]thiazol-2-yl)acetonitrile (S2): S2 was synthesized according to literature method.[S1] To a solution of 2-aminothiophenol (**S1**, 0.79 mL, 7.57 mmol) in 6 mL of ethanol was added malononitrile (1.00 g, 15.13 mmol) in 1 mL of glacial acetic acid. The reaction mixture was stirred for 12 h at rt. The resultant yellow precipitate was collected by filtration, followed by washing with 200 mL of distilled water. Compound **S2** (2.58 g) was thus obtained in 98% yield. R_f = 0.50 (EtOAc/hexane =1/7). ^{1}H NMR ($CDCl_3$, 400 MHz): δ 8.05 (d, J = 8.2 Hz, 1 H), 7.91 (d, J = 8.1 Hz, 1 H), 7.54 (td, J = 7.3, 1.2 Hz, 1 H), 7.46 (td, J = 8.1, 1.2 Hz, 1 H), 4.25 (s, 2 H).

2-(1,3-Dithian-2-yl)-4-nitrophenol (S4): S4 was synthesized according to literature method.[S2] To an ice-cooled solution of 2-hydroxy-5-nitrobenzaldehyde (**S3**, 1.0 g, 5.98 mmol) and 1,3-propanedithiol (0.79 mL, 7.77 mmol) in anhydrous CH_2Cl_2 (30 mL) was dropwise added BF_3•OEt_2 (1.11 mL, 8.97 mmol). After addition, the reaction mixture was warmed up to room

temperature and stirred for 2 h. It was then quenched with MeOH and evaporated to dryness to give the crude product. The residue was recrystallized in EtOAc/hexane, and pure **S4** was obtained as a yellow solid in high yield (1.46 g, 95%). R_f = 0.52 (EtOAc/hexane = 1/1). ^{1}H NMR ($CDCl_3$, 400 MHz): δ 8.26 (d, *J* = 2.8 Hz, 1 H), 8.14 (dd, *J* = 9.0, 2.8 Hz,1 H), 6.99 (d, *J* = 9.1 Hz, 1 H), 5.42 (s, 1 H), 3.09 (td, *J* = 14.6, 2.6 Hz, 2 H), 2.97 (dt, *J* = 14.6, 4.1 Hz, 2 H), 2.28-2.19 (m, 1 H), 2.04-1.90 (m, 2 H).

5-Azido-2-hydroxybenzaldehyde (S5): S5 was synthesized according to literature method.[S2] Zinc (1.86 g, 28.4 mmol) was added to a solution of **S4** (1.46 g, 5.68 mmol) in 30 mL of AcOH. The reaction mixture was stirred for 1 h at rt. It was then flitered through celite and evaporated to dryness. The residue was dissolved in AcOH (30 mL) and cooled to 0 °C. An aqueous solution of $NaNO_2$ (1.50 g, 22.7 mmol) was added into the reaction and the mixture was stirred for 2 h. At this time, a solution of NaN_3(aq) (554 mg, 8.52 mmol) was dropwise added to the reaction mixture. After addition, the reaction was stirred for 30 min. The solvent was then removed and the residue was dissolved in the EtOAc (50 mL). The organic phase was washed sequentially with saturated $NaHCO_3$(aq) (50 mL × 3), H_2O (50 mL × 2), and brine (50 mL). The organic layer was dried over anydrous Na_2SO_4, filtered, and concentrated to give the crude product. The desired product **S5** was purified with silica gel column chromatography eluented with EtOAc/hexane (1/7). Compound **S5** was obtained as a yellow solid in 80% yield (741 mg). R_f = 0.45 (EtOAc/hexane = 1/7). ^{1}H NMR ($CDCl_3$, 400 MHz): δ 10.85 (s, 1 H), 9.88 (s, 1 H), 7.24-7.19 (m, 2 H), 7.02 (d, *J* = 9.9 Hz, 1 H).

6-Azido-3-(benzo[d]thiazol-2-yl)-2H-chromen-2-imine (13): Piperidine (60 μL, 0.61 mmol) was added to a solution of compound **S2** (100 mg, 0.61 mmol) and compound **S5** (106 mg, 0.61 mmol) in 6 mL of EtOH. The mixture was stirred for 1 h at rt. Acetic acid (70 μL, 1.22 mmol) was

added to the mixture and the solution was stirred for another hour. The resultant precipitate was collected by filtration, and washed with EtOH (10 mL × 3) to afford compound **13**. Compound **13** was obtained as a yellow solid in 86% yield (168 mg). R_f = 0.60 (EtOAc/hexane =1/1), mp = 190-193 °C. ^{1}H NMR (DMSO-d_6, 400 MHz): δ 9.17 (s, 1 H), 8.79 (s, 1 H), 8.16 (d, J = 7.8 Hz, 1 H), 8.07 (d, J = 7.8 Hz, 1 H), 7.71 (s, 1 H), 7.57 (t, J = 7.3 Hz, 1 H), 7.47 (t, J = 7.3 Hz, 1 H), 7.26 (m, 2 H). ^{13}C NMR (DMSO-d_6, 100 MHz): δ 160.4 (C), 152.9 (C), 151.7 (C), 150.3 (C), 137.0 (C), 135.1 (C), 134.5 (C), 126.4 (CH), 125.2 (CH), 122.9 (CH), 122.5 (CH), 122.0 (CH), 120.0 (CH), 119.3 (CH), 116.5 (CH). IR (KBr): 3235, 2915, 2850, 2116, 1735, 1718, 1705, 1701, 1685, 1660, 1650, 1638, 1490, 1472, 1318, 1226, 963, 939, 870, 803, 754, 737, 726 cm^{-1}. HR-ESI MS (*m/z*) calcd for $C_{16}H_{10}N_5OS$ $(M+H)^+$ 320.0601, and found 320.0602.

Synthesis of compounds 14 and 15

7-((5a*R*,6*R*,6a*S*)-6-(Hydroxymethyl)-5,5a,6,6a,7,8-hexahydrocyclopropa[5,6]cycloocta[1,2-*d*][1,2,3]triazol-1(4*H*)-yl)-4-methyl-2*H*-chromen-2-one (14): A reaction solution was prepared by mixing compound **11** (30 mg, 0.20 mmol) with compound **12** (36 mg, 0.18 mmol) in 0.5 mL of CH_2Cl_2. The reaction was stirred at rt for 12 h. The solvent was removed, and the residue was purified with silica gel column chromatography to give compound **14** in 74% yield (47 mg) as a white solid. R_f = 0.40 (EtOAc), mp = 99-101 °C. ^{1}H NMR ($CDCl_3$, 400 MHz): δ 7.77 (d, J = 8.4 Hz, 1 H), 7.41 (dd, J = 8.4, 2.1 Hz, 1 H), 7.36 (d, J = 2.0 Hz, 1 H), 6.38 (d, J = 1.2 Hz, 1 H), 4.68 (s, 1 H), 3.48-3.35 (m, 2 H), 3.24-3.15 (m, 1 H), 3.00-2.87 (m, 1 H), 2.75-2.65 (m, 1 H), 2.54-2.36 (m, 5 H), 1.50-1.37 (m, 2 H), 0.95-0.77 (m, 2 H), 0.76-0.67 (m, 1 H). ^{13}C NMR (DMSO-d_6, 100 MHz): δ 159.9 (C), 153.8 (C), 151.6 (C), 146.2 (C), 139.2 (C), 134.6 (C), 125.9 (CH), 121.6 (CH), 120.7 (C), 116.3 (CH), 114.2 (CH), 94.6 (CH_2), 71.2 (CH_2), 27.6 (CH_2), 27.3 (CH_2), 25.8 (CH), 25.3 (CH_2), 23.5 (CH_2), 22.7 (CH), 18.8 (CH_3). IR (KBr): 3468, 2989, 2923, 2859, 1740, 1616,

1570, 1511, 1435, 1390, 1366, 1264, 1240, 1170, 1105, 1043, 1008, 943, 862, 731, 646 cm^{-1}. HR-ESI MS (*m/z*) calcd for $C_{20}H_{22}N_3O_3$ $(M+H)^+$ 352.1656, and found 352.1633.

((5aR,6aS)-1-(3-(Benzo[d]thiazol-2-yl)-2-imino-2H-chromen-6-yl)-1,4,5,5a,6,6a,7,8-octahydrocyclopropa[5,6]cycloocta[1,2-d][1,2,3]triazol-6-yl)methanol (15): A reaction solution was prepared by mixing compound **11** (10 mg, 0.06 mmol) with compound **13** (10 mg, 0.03 mmol) in 0.5 mL of MeOH. The reaction was stirred at rt for 12 h. It was then poured into ether. The precipitates thus formed were collected to afford the **15** as yellow solid in 74% yield (10 mg, 0.02 mmol). R_f = 0.40 (EtOAc/hexane = 4/1), mp = 95-98 °C. ^{1}H NMR ($CDCl_3$, 400 MHz): δ 9.16 (s, 1 H), 8.13 (d, *J* = 8.2 Hz, 1 H), 8.02 (d, *J* = 8.2 Hz, 1 H), 7.79 (s, 1 H), 7.67 (d, *J* = 7.5 Hz, 1 H), 7.62 (d, *J* = 7.6 Hz , 1 H), 7.57 (t, *J* = 7.5 Hz, 1 H), 7.48 (t, *J* = 7.6 Hz, 1 H), 3.61-3.46 (m, 2 H), 3.32-3.21 (m, 1 H), 3.05-2.87 (m, 2 H), 2.79-2.66 (m, 1 H), 2.59-2.48 (m, 1 H), 1.52-1.40 (m, 2 H), 1.00-0.75 (m, 3 H). ^{13}C NMR (DMSO-d_6, 100 MHz): δ 159.2 (C), 153.4 (C), 152.0 (C), 144.6 (C), 141.2 (C), 136.1 (C), 134.9 (C), 133.0 (CH), 126.9 (CH), 125.8 (CH), 122.8 (CH), 122.4 (CH), 120.8 (CH), 119.6 (CH), 117.8 (CH), 77.3 (CH_2), 76.8 (CH_2), 76.0 (CH_2), 65.8 (CH_2), 64.0 (CH_2), 62.9 (CH_2), 27.6 (CH), 26.9 (CH_2), 26.4 (CH_2), 25.4 (CH_2), 22.9 (CH_2), 21.5 (CH_2). IR (KBr): 3235, 2915, 2850, 1735, 1718, 1705, 1701, 1685, 1660, 1650, 1638, 1490, 1472, 1318, 1226, 963, 939, 870, 803, 754, 737, 726 cm^{-1}. HR-ESI MS (*m/z*) calcd for $C_{26}H_{24}N_5O2S$ $(M+H)^+$ 470.1645, and found 470.1637.

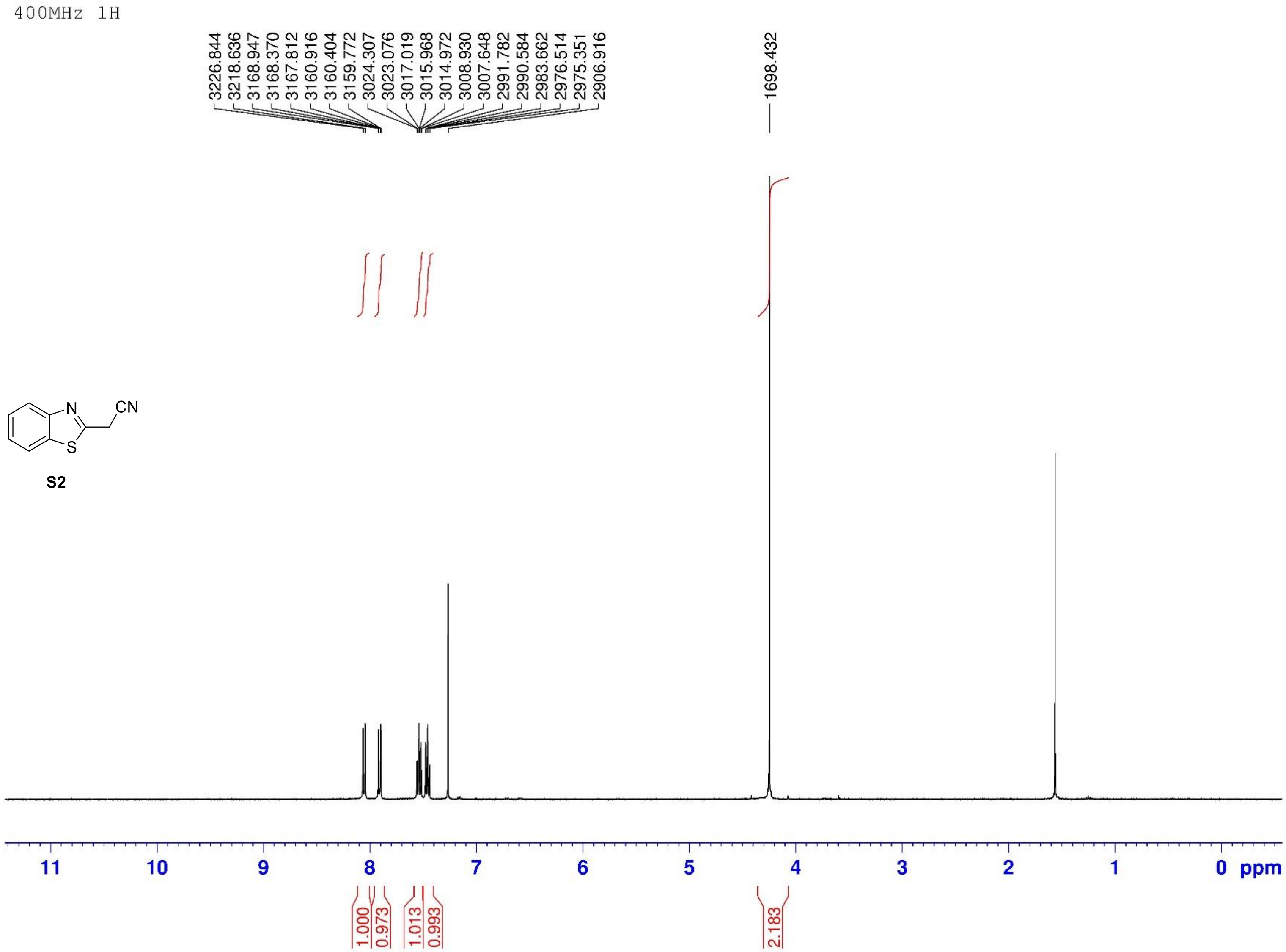

Figure S2. ^{1}H NMR spectrum of compound **S2** (400 MHz, $CDCl_3$).

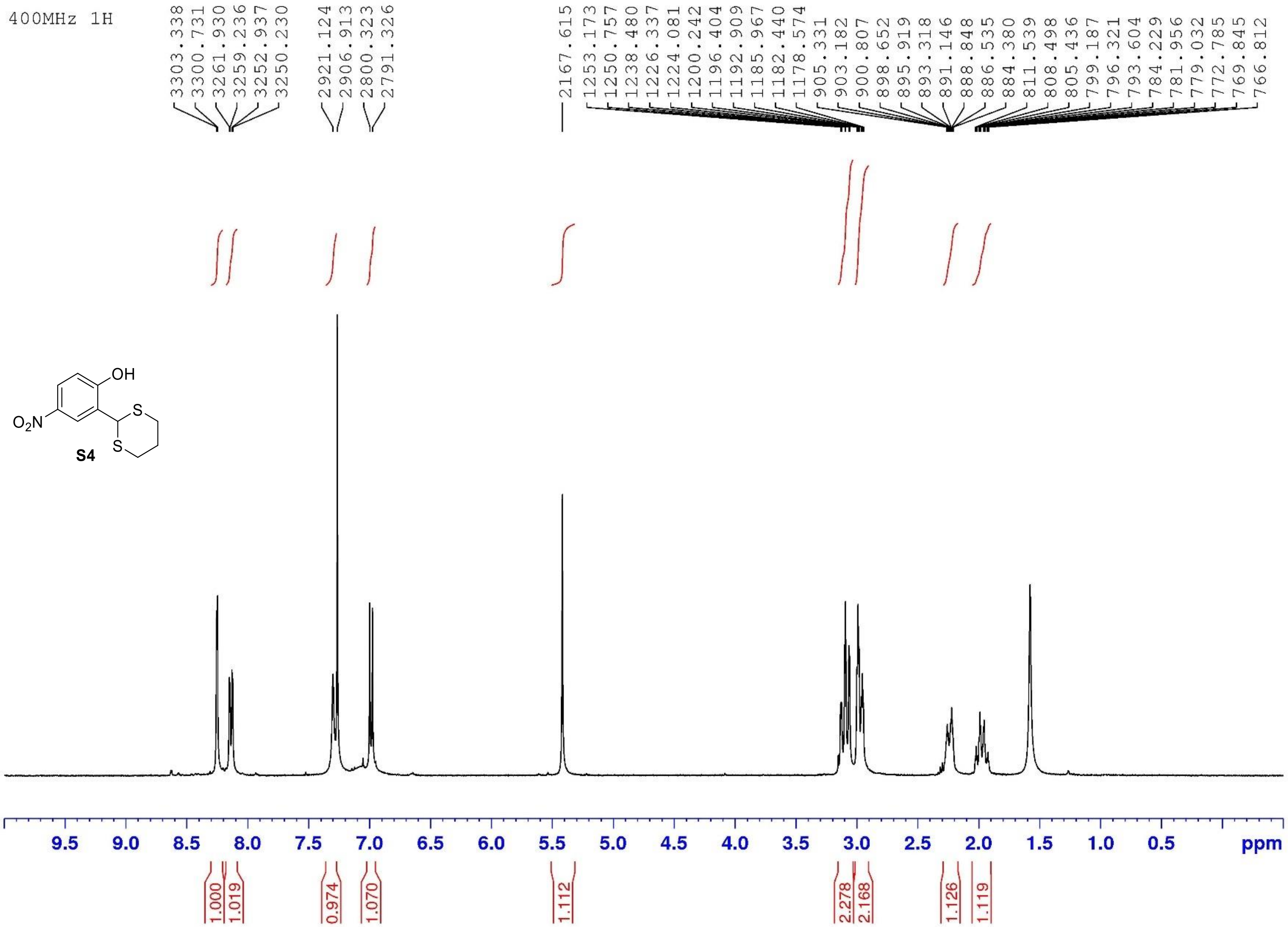

Figure S3. ^{1}H NMR spectrum of compound **S4** (400 MHz, $CDCl_3$).

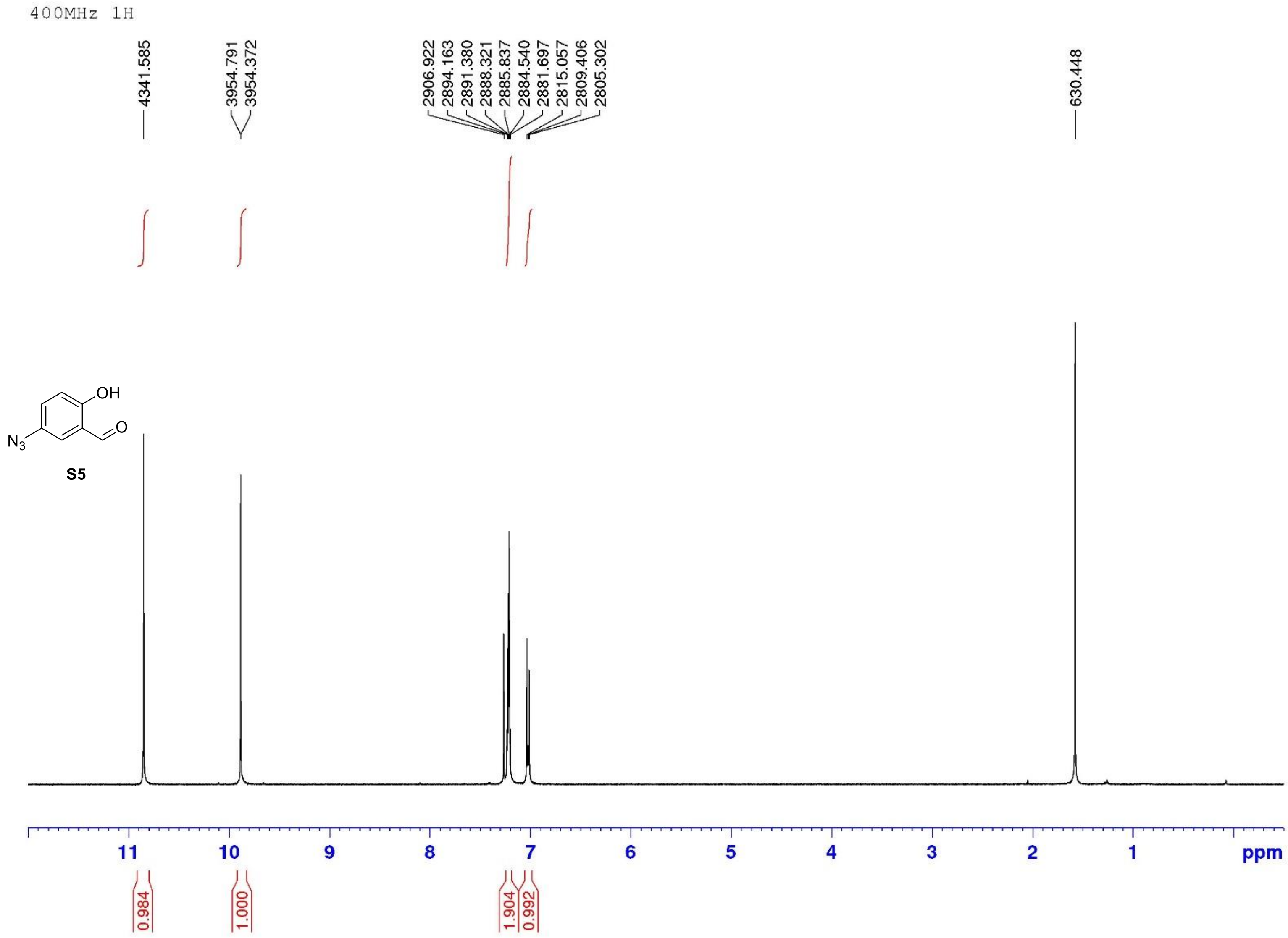

Figure S4. ^{1}H NMR spectrum of compound **S5** (400 MHz, $CDCl_3$).

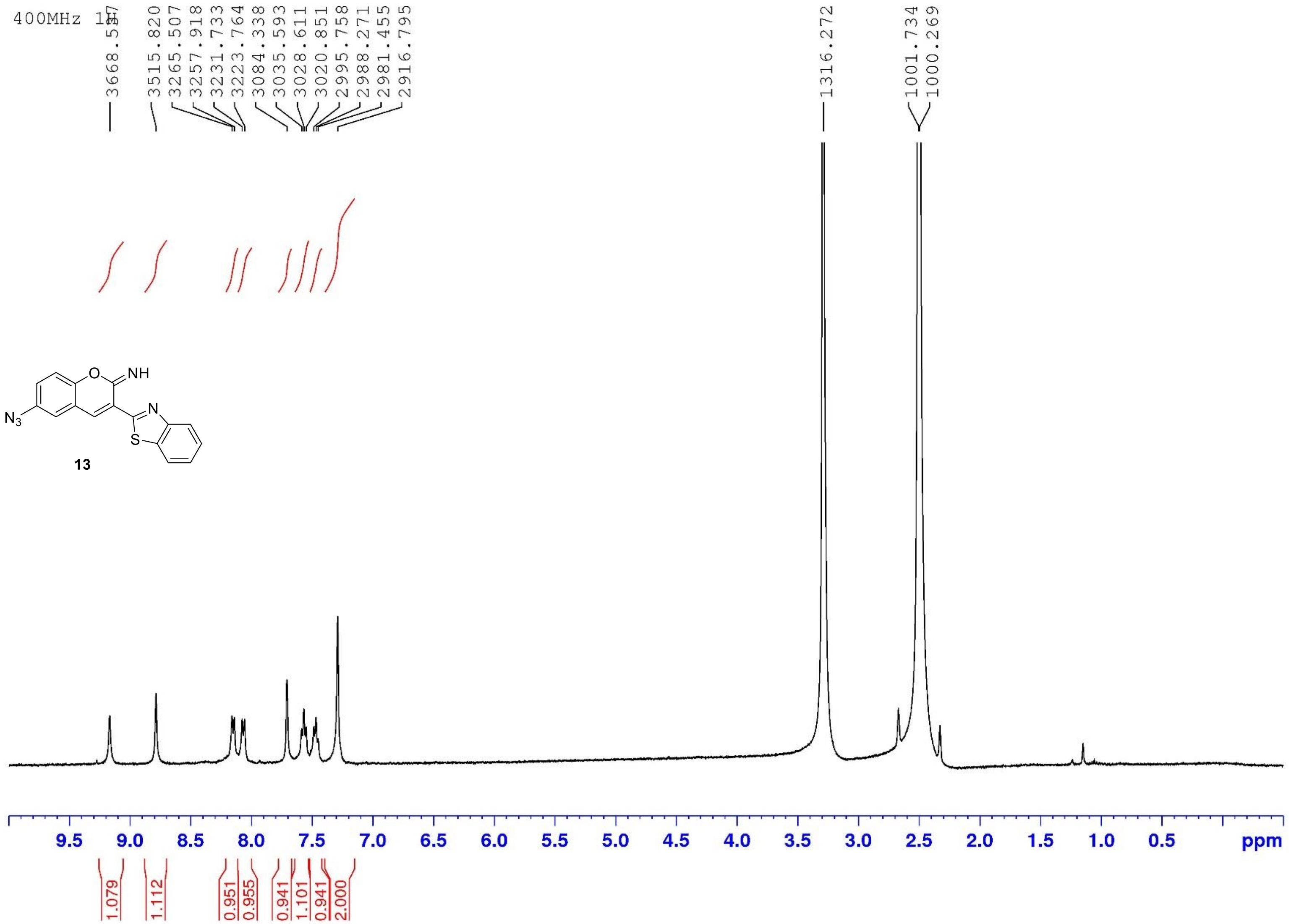

Figure S5. ^{1}H NMR spectrum of compound **13** (400 MHz, DMSO-d_6).

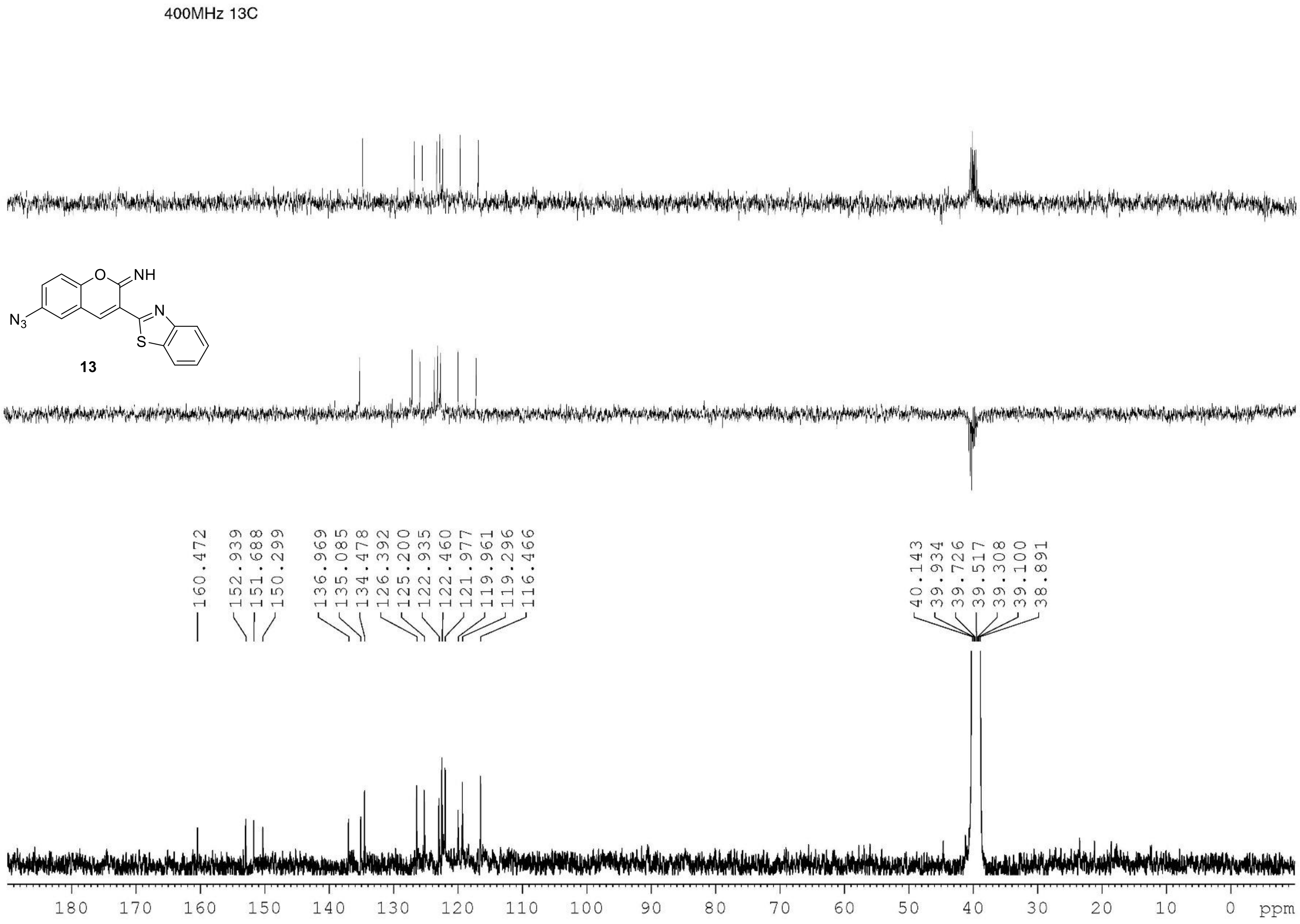

Figure S6. ^{13}C NMR spectrum of compound **13** (100 MHz, DMSO-d_6).

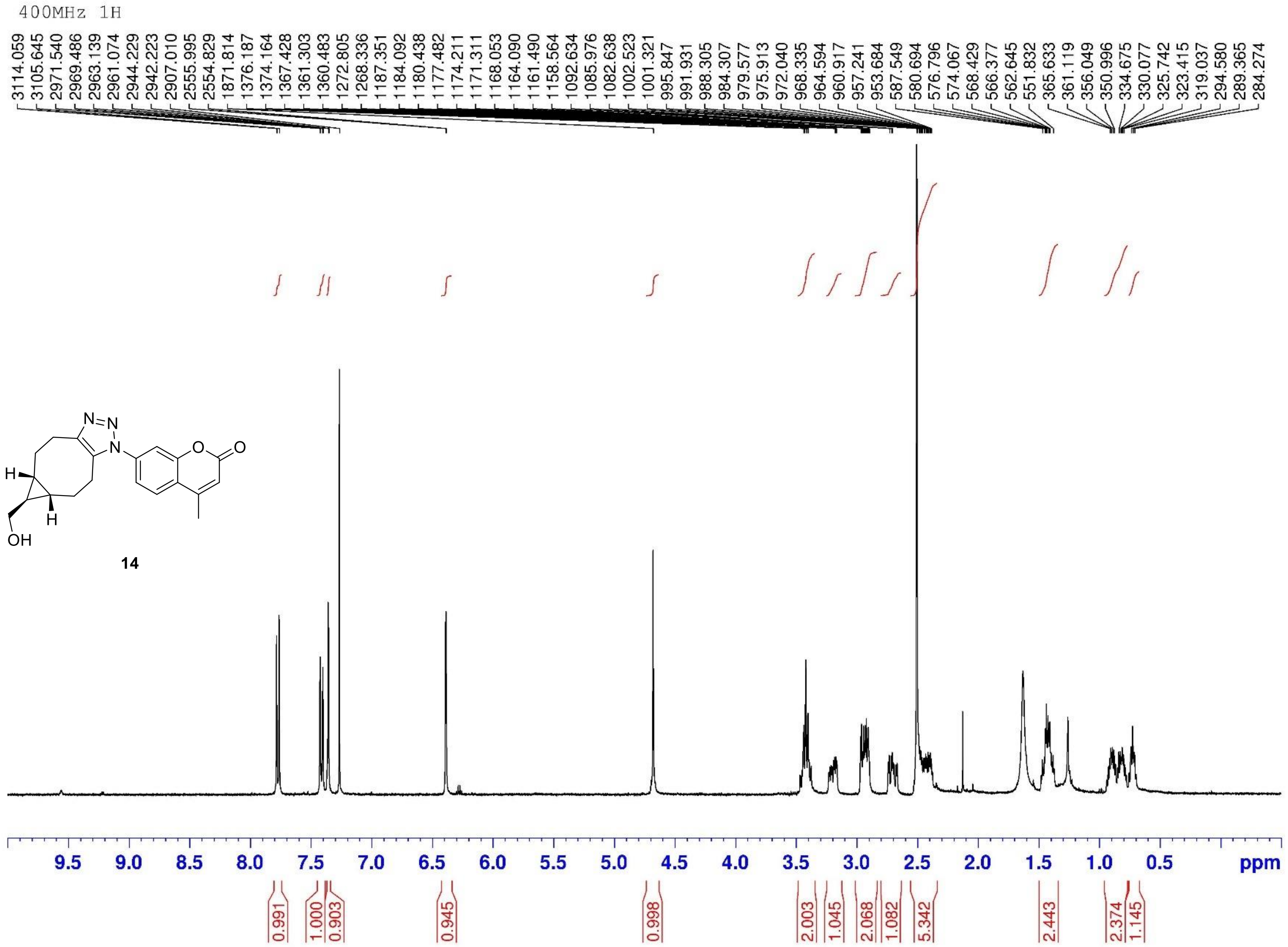

Figure S7. ¹H NMR spectrum of compound **14** (400 MHz, $CDCl_3$).

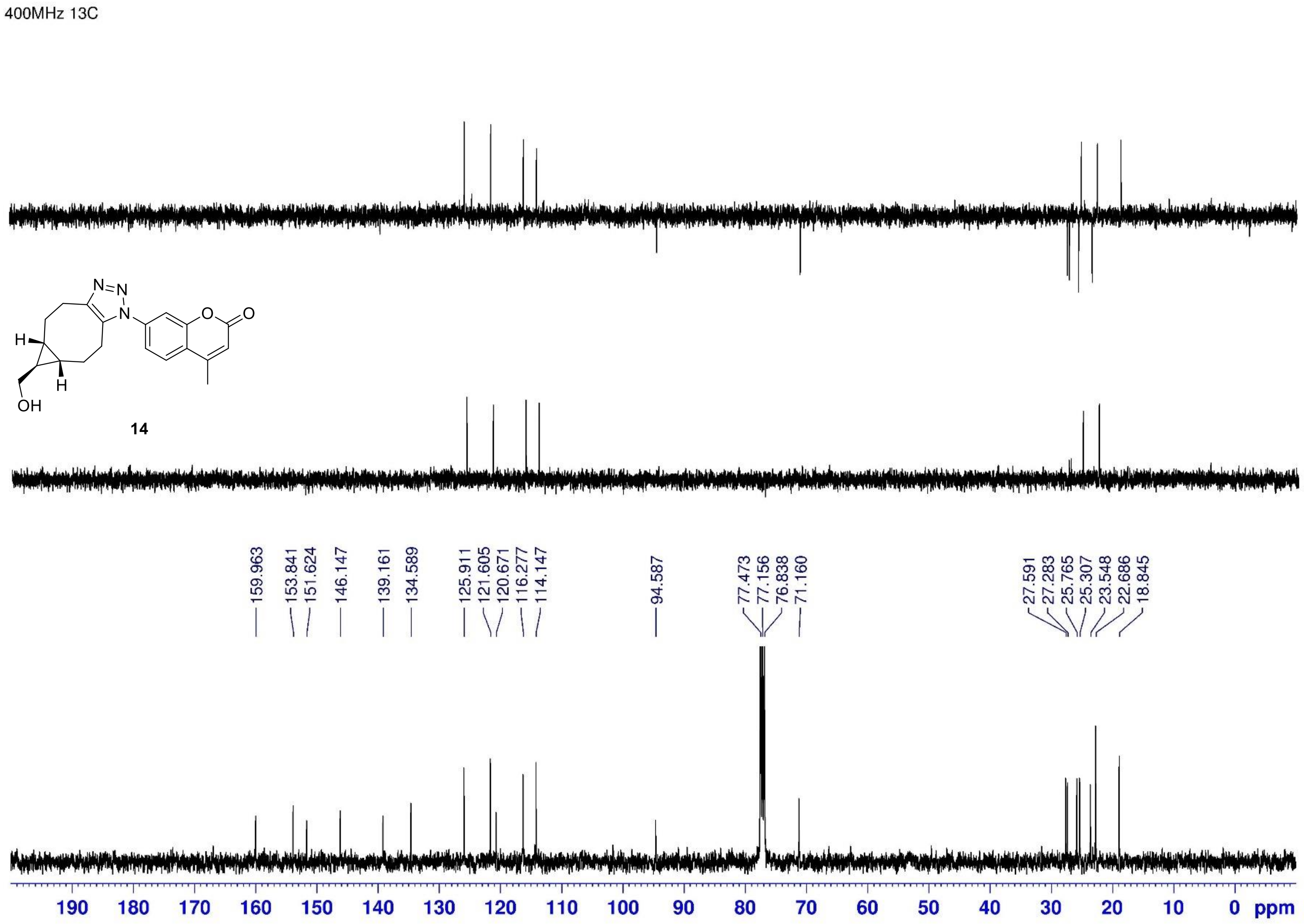

Figure S8. ^{13}C NMR spectrum of compound **14** (400 MHz, $CDCl_3$).

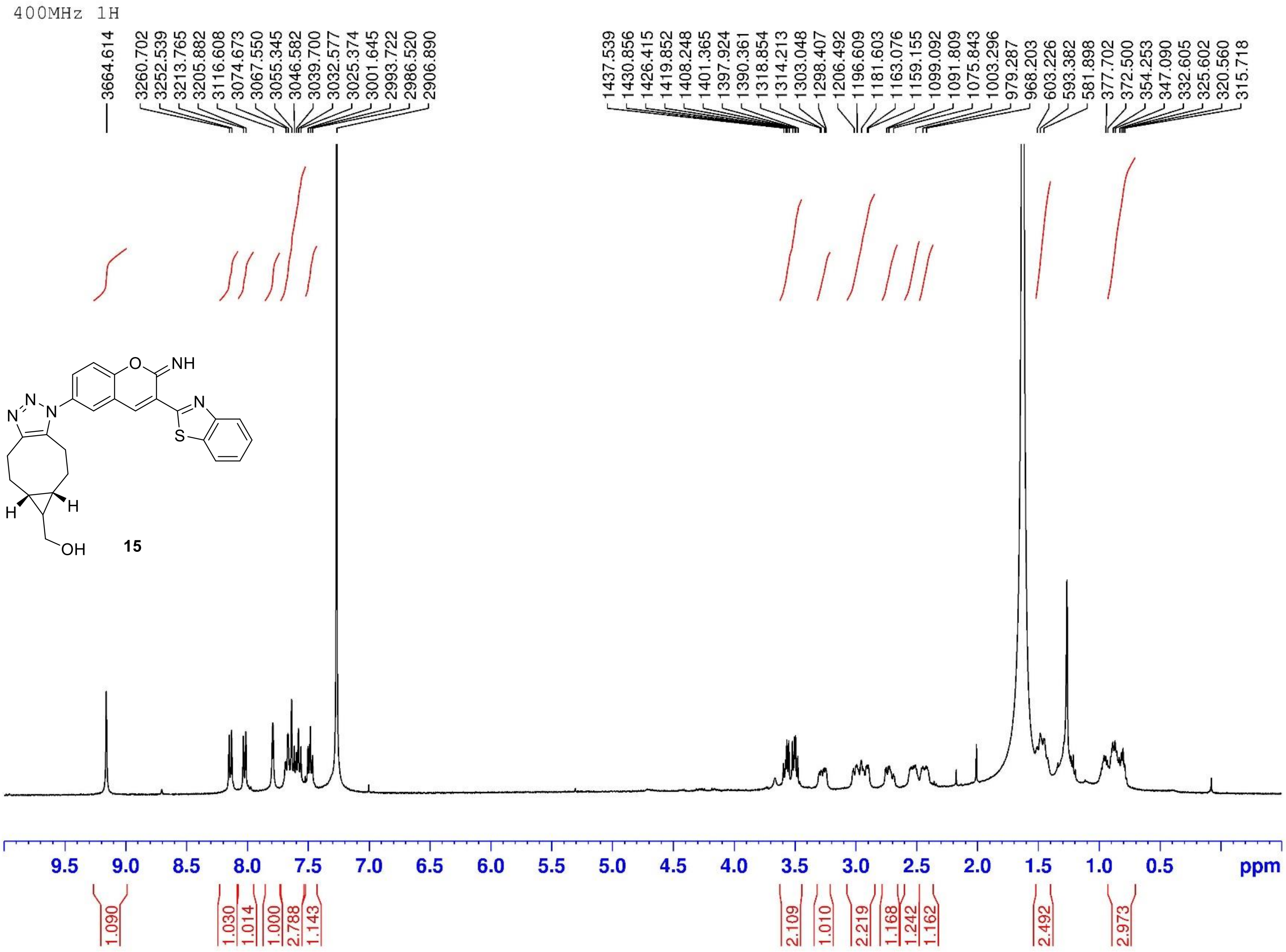

Figure S9. ^{1}H NMR spectrum of compound **15** (400 MHz, $CDCl_3$).

400MHz 13C

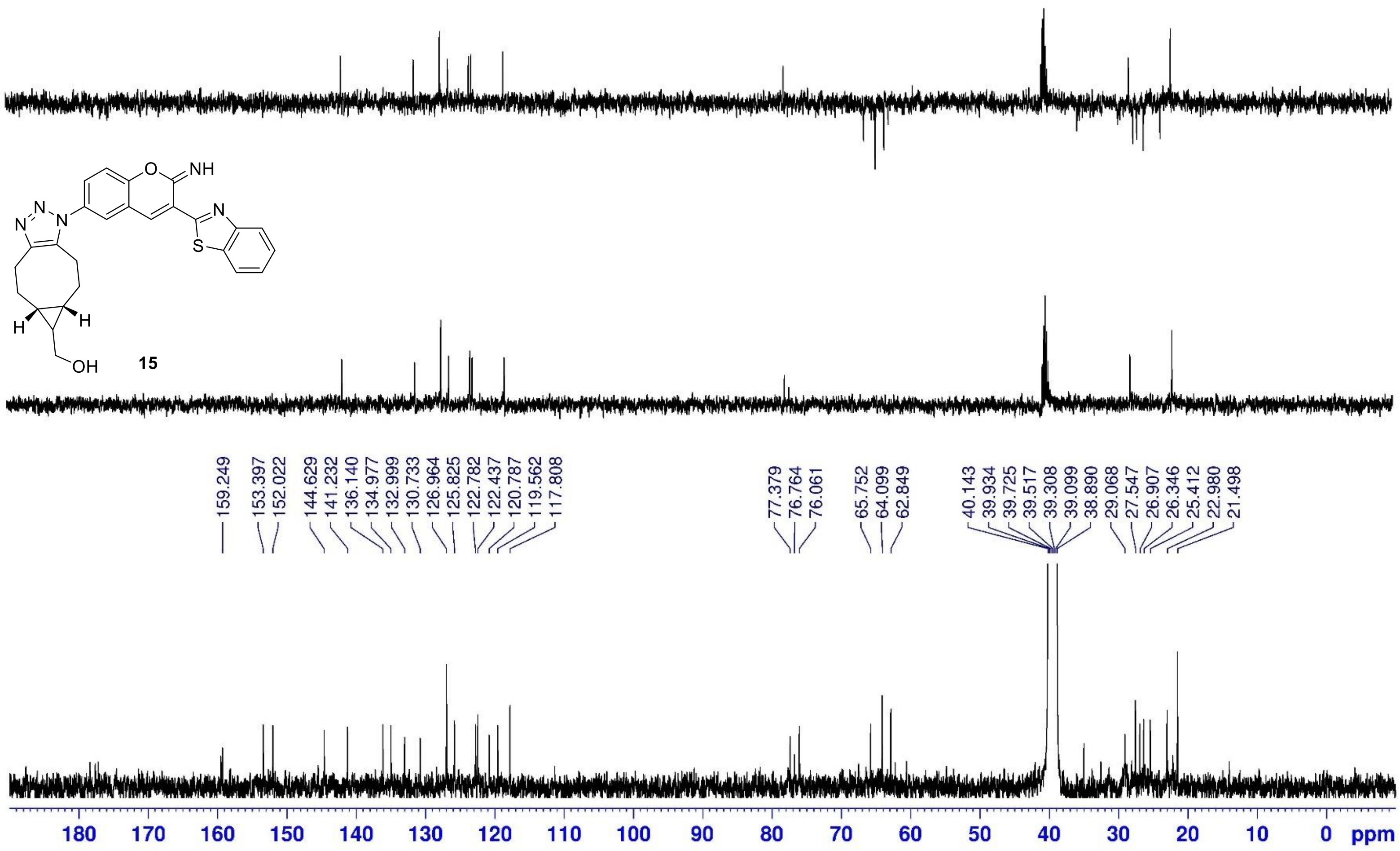

Figure S10. ^{13}C NMR spectrum of compound **15** (100 MHz, DMSO-d_6).

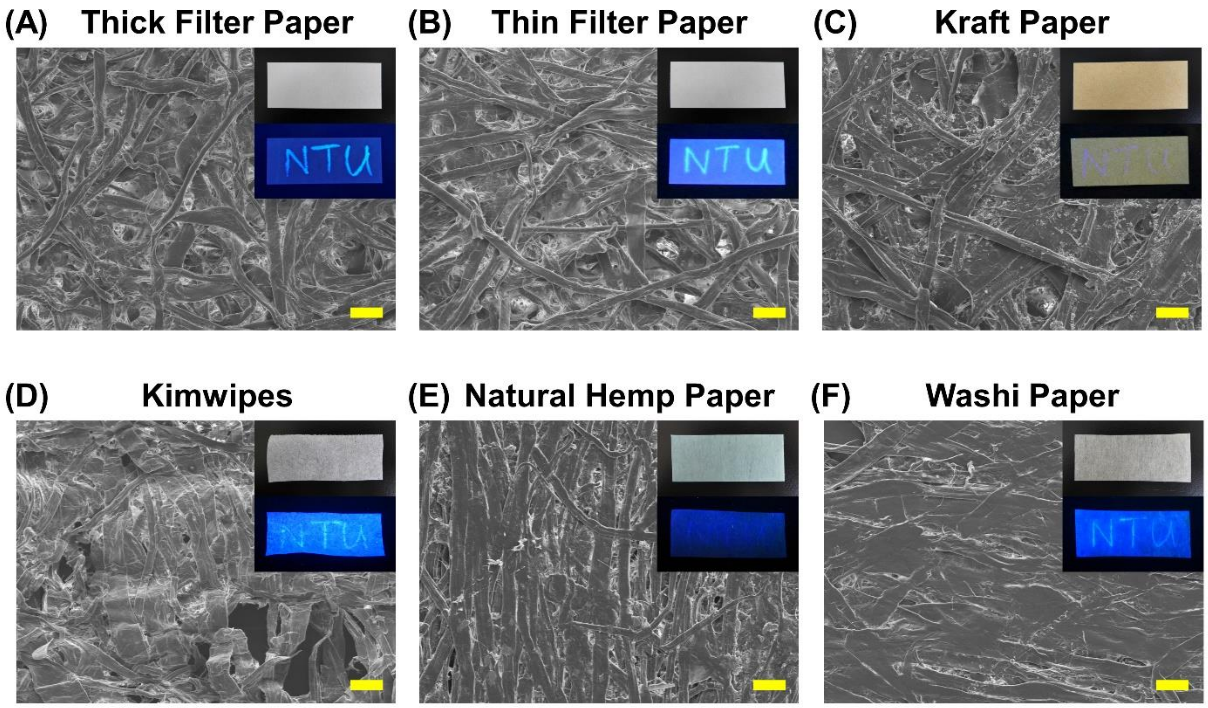

Figure S11. Effects of different paper types. SEM images of pristine paper substrates: (A) thick filter paper, (B) thin filter paper, (C) kraft paper, (D) Kimwipes, (E) natural hemp paper, and (F) Washi paper. (B) and (C): Paper materials with medium pore size and lower surface hydrophobicity. (E) and (F): Paper materials with smaller pore size and higher surface hydrophobicity. The scale bars are 500 µm. Inset: Photographs of pristine paper (top) and the SPAAC click reaction resulted fluorescent pattern (bottom).

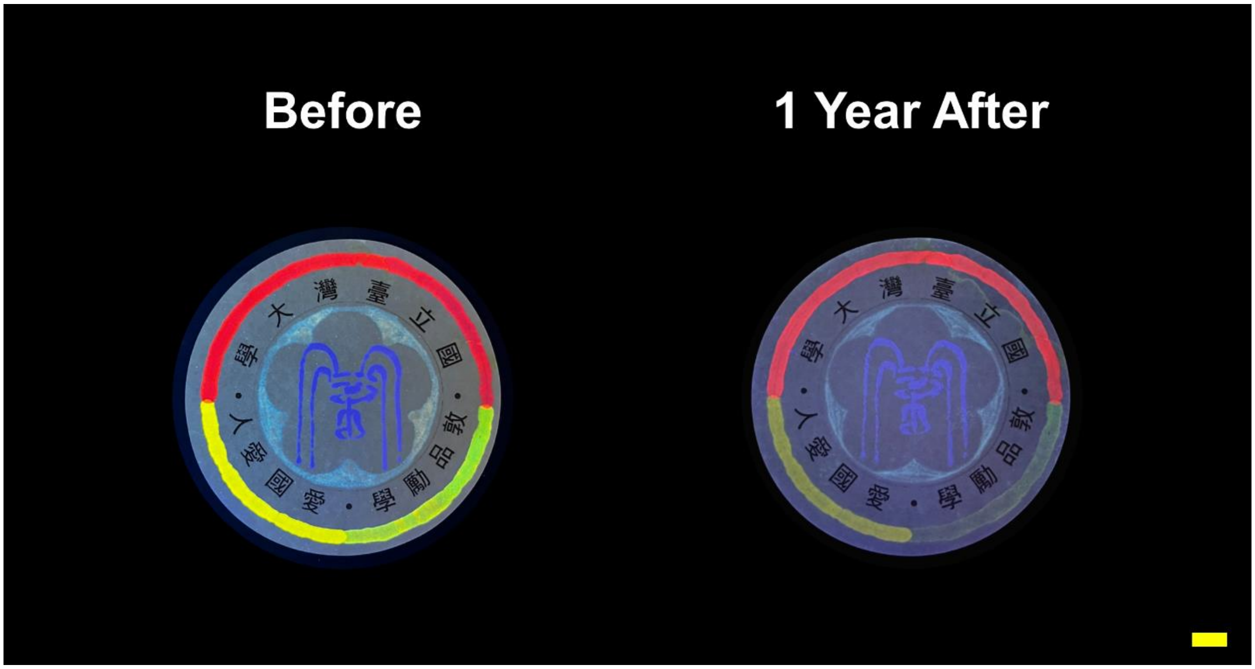

Figure S12. Shelf life test of the reaction products on this platform. The fluorescence contrast slightly reduces after one year of storage due to the natural decay of organic dyes. The scale bar is 1 cm.

References

S1. AlNeyadi, S. S.; Salem, A. A.; Ghattas, M. A.; Atatreh, N.; Abdou, I. M., Antibacterial activity and mechanism of action of the benzazole acrylonitrile-based compounds: In vitro, spectroscopic, and docking studies, *Eur. J. Med. Chem.*, **2017**, *136*, 270-282.

S2. Kai, H.; Hinou, H.; Nishimura, S.-I., Aglycone-focused randomization of 2-difluoromethylphenyl-type sialoside suicide substrates for neuraminidases, *Bioorg. Med. Chem.*, **2012**, *20*, 2739-2746.
